# Double-Walled Mesoporous Hydrogen-Bonded Organic Frameworks with High Methane Storage Capacity

**DOI:** 10.1021/jacs.5c02705

**Published:** 2025-04-29

**Authors:** Ruihua Zhang, Chun Tang, Shuliang Yang, Penghao Li, Han Han, Yong Wu, Guangcheng Wu, Xueze Zhao, Bai-Tong Liu, Sheng-Nan Lei, Bohan Tang, Enxu Liu, Yi-Kang Xing, Charlotte L. Stern, Christos D. Malliakas, J. Fraser Stoddart

**Affiliations:** † Department of Chemistry, The University of Hong Kong, Hong Kong SAR 999077, China; ‡ Department of Chemistry, Northwestern University, 2145 Sheridan Road, Evanston, Illinois 60208, United States; § College of Energy, College of Chemistry and Chemical Engineering, Xiamen University, Xiamen, Fujian 361005, China; ∥ Department of Chemistry, The University of Mississippi, University, Mississippi 38677, United States; ⊥ Center for Regenerative Nanomedicine, Northwestern University, 303 East Superior Street, Chicago, Illinois 60611, United States; # Stoddart Institute of Molecular Science, Department of Chemistry, Zhejiang University, Hangzhou 310027, China; ∇ ZJU-Hangzhou Global Scientific and Technological Innovation Center, Hangzhou 311215, China; ○ School of Chemistry, University of New South Wales, Sydney, New South Wales 2052, Australia

## Abstract

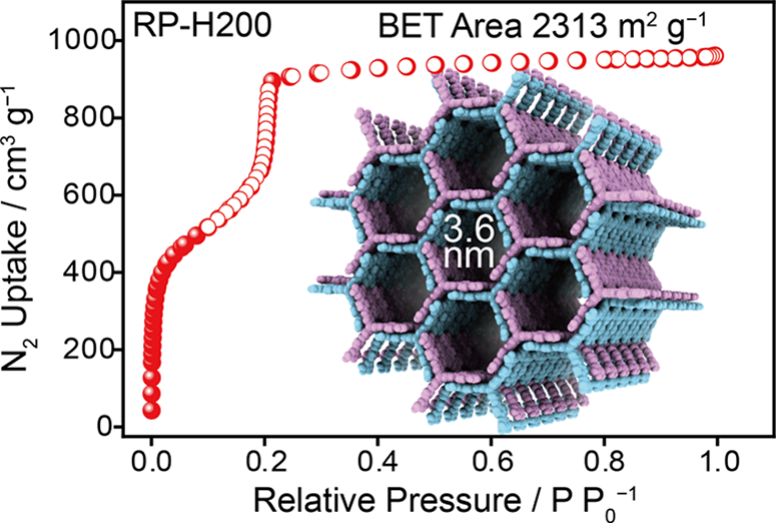

The development of mesoporous hydrogen-bonded organic frameworks (HOFs) is critically important for various applications, yet it poses significant challenges. Herein, we present the synthesis and characterization of a robust mesoporous HOF, RP-H200, constructed through the orchestration of π–π stacking and hydrogen bonding interactions in a 2-fold interpenetrated framework. RP-H200 features a unique double-walled structure with a pore size of 3.6 nm, representing the largest pore size among reported HOFs to date. The framework exhibits a high surface area of 2313 m^2^ g^–1^, with aromatic surfaces dominating the mesoporous channels. The methane storage performance of RP-H200 reaches a high capacity of 0.31 g g^–1^ at 270 K/100 bar and 0.25 g g^–1^ at 296 K/100 bar. The combination of large permanent mesoporosity, excellent thermal stability, and high surface area in RP-H200 makes it a promising candidate for clean energy storage and other functions.

## Introduction

Mesoporous materials (pore sizes ranging from 2 to 50 nm) have gained significant interest because of their broad application scenarios,^1,2^ including adsorption, separation, energy storage and catalysis, etc. Framework materials,^3−10^ such as metal–organic frameworks (MOFs) and covalent organic frameworks (COFs), have shown the feasibility of constructing periodic mesopores using bottom-up approaches, offering^11−13^ advantages of structural diversity and functional tunability. Hydrogen-bonded organic frameworks (HOFs) represent^14−17^ an emerging class of framework materials constructed from the assembly of small molecular building blocks through hydrogen bonding interactions, along with other noncovalent interactions. As HOFs are essentially a type of molecular crystal, they not only feature^18−23^ the mentioned advantages of MOFs and COFs but also offer^16,17,24−27^ notable solution-processability and recyclability.

**Figure 1:**
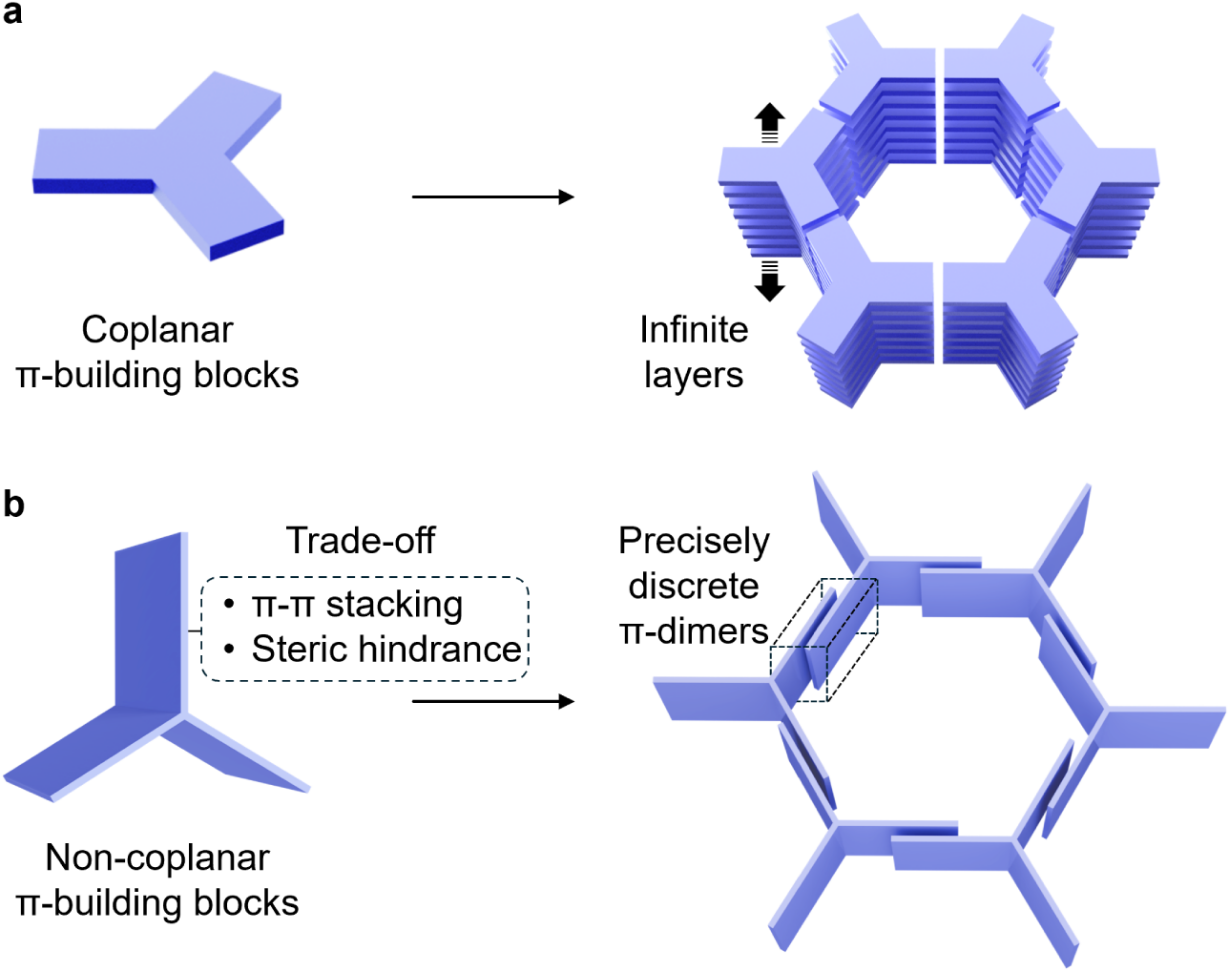
Strategies for the construction of mesoporous HOFs. (a) Coplanar π-building blocks tend to stack with adjacent molecules, with both sides of the aromatic rings participating, resulting in infinite π–π stacks in framework materials. This framework type features channel surfaces dominated by the edges of the aromatic rings. (b) A trade-off between π–π stacking and steric hindrance occurs when noncoplanar π-building blocks are employed, leading to the formation of precisely discrete π-dimers, as illustrated in dashed frame. In this situation, only one side of the aromatic rings engages in π–π stacking with neighboring molecules, while the opposite side remains exposed to the channel surface.

Considerable efforts have been devoted to the advancement of HOFs, leading to the development of numerous microporous HOFs that have demonstrated promising applications in areas such as gas separation and storage,^6,7,28−31^ proton conduction,^32,33^ sensing^34,35^ and heterogeneous catalysis.^36,37^ Despite these advancements, the construction of mesoporous HOFs with permanent porosity remains a challenging endeavor. Molecular assembly^38,39^ tends to maximize noncovalent interactions, which typically result in the formation of densely packed structures, making the formation and stabilization of large mesopores in HOFs difficult.^40^ Recently, the utilization of coplanar π-building blocks (Figure 1a) has been demonstrated^41−46^ to be a successful strategy for constructing HOFs with permeant mesoporosity. Examples include PFC-2^43^ constructed from 1,3,6,8-tetrakis(*p*-benzoic acid)pyrene with a pore diameter approaching 3.0 nm, along with HOF-14 and HOF-102 assembled from 1,3,6,8-tetra(6-carboxynaphthalen-2-yl)pyrene,^44,45^ featuring a large pore size of 2.4 × 3.1 nm.

It is notable that nearly all reported mesoporous HOFs exhibiting permanent mesoporosity are constructed from coplanar π-building blocks (Figure 1a), which leads to a shared common feature—the formation of infinite π–π stacks^47^ during the layer-to-layer assembly process. Such infinite π–π stacking facilitates efficient interactions among adjacent molecular building blocks within the frameworks, which, however, inevitably hinders the efficient utilization of molecular surfaces. At the same time, the exposed channel surfaces of the reported mesoporous HOFs are dominated by the edges of aromatic rings. Therefore, there is a compelling need to develop mesoporous HOFs with pores or channels exposing more aromatic surfaces,^23,28,48^ which would not only diversify the pore chemistry but also broaden their application scenarios, serving as critical complements to the currently reported HOFs.

In this research, we employed (Figure 1b) noncoplanar π-building blocks to construct mesoporous HOF, rather than using coplanar π-building blocks. The noncoplanar configuration introduces a trade-off between π–π stacking and steric hindrance, facilitating the formation of precisely discrete π-dimers where only one side of each wing participates in π–π stacking. This contrasts with the coplanar system (Figure 1a), which tends to form infinite π–π stacks involving both sides of the building blocks. It is worth noting that the noncoplanar π-building block could potentially change its conformation to coplanar arrangement, leading to the formation of infinite π–π stacks. Therefore, controlling the noncoplanar conformation (Figure 1b) during the assembly of the building blocks is crucial. Utilizing this strategy, a mesoporous HOF designated as RP-H200 was successfully synthesized, which features a rare double-walled framework structure with a pore size of 3.6 nm, the largest among all reported HOFs so far. Furthermore, RP-H200 exhibits a surface area of 2313 m^2^ g^–1^ and a unique channel chemistry dominated by aromatic surfaces, which endows it with high storage capacities for clean energy gases, including methane and hydrogen. Specifically, RP-H200 demonstrates a methane storage capacity of 0.31 g g^–1^ at 270 K/100 bar and 0.25 g g^–1^ at 296 K/100 bar, as well as a hydrogen storage capacity of 6.7 wt % (36.6 g L^–1^) at 77 K/100 bar. The combination of large pore size, robust thermal stability, and high surface area in RP-H200 makes it a promising material for various applications.

## Results and Discussion

### Synthesis and Structural Analysis of the Framework

**Figure 2:**
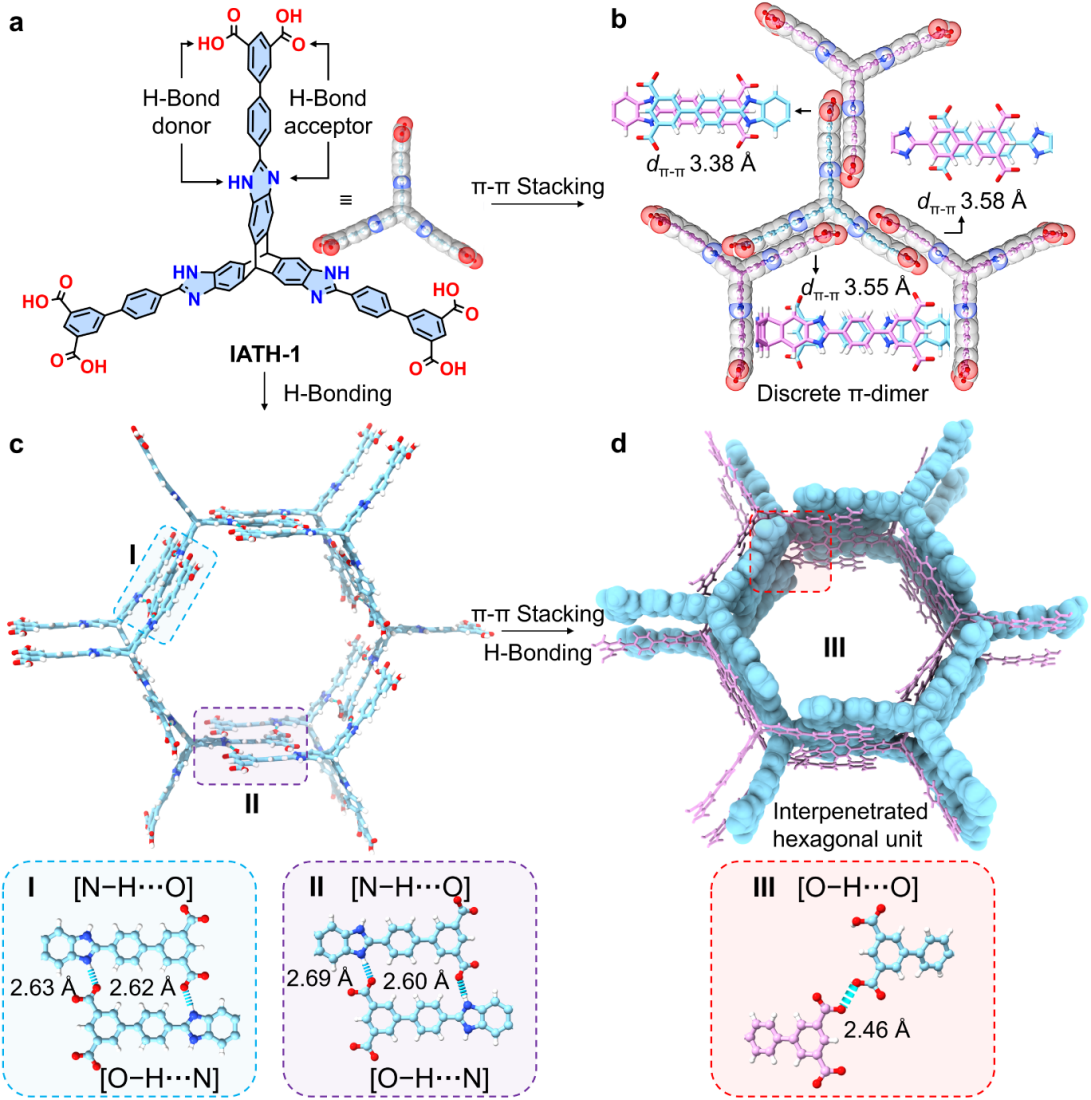
Noncovalent interaction analysis of the single-crystal superstructures of RP-H200. (a) Structural formula of IATH-1. Each wing of IATH-1 contains two types of hydrogen-bond donors and two types of hydrogen-bond acceptors, respectively. The top view of IATH-1 is represented by the stick model, with the corresponding semitransparent space-filling models overlaid on it. (b) The top view of representatively discrete π-dimers in RP-H200, composed of four IATH-1 through π–π stacking. The details of π–π stacking between two wings are shown in the stick model with one wing shown in light blue and the other wing in magenta. (c) A hexagonal unit formed by nine IATH-1 molecules through hydrogen bonding interactions is shown in the stick model. Regions I and II show the detailed views of [N–H···O] and [O–H···N] hydrogen bonding interactions. (d) Two hexagonal units interpenetrate with each other. Region III details one of the [O–H···O] hydrogen bonding interactions between the two interpenetrated hexagonal units.

In order to implement the strategy illustrated in Figure 1b, we utilized^30^ (Figure 2a) a noncoplanar π-building block, imidazole-annulated triptycene hexaacid (IATH-1), to construct the mesoporous HOF, RP-H200. IATH-1 is a triptycene-based^49,50^ molecule with three identical wings, each featuring two types of hydrogen-bond donors and acceptors: oxygen atoms (in red) from the carboxyl groups and nitrogen atoms (in blue) from the imidazole groups. The conformation of each wing can achieve a good coplanarity among aromatic rings, which is beneficial for π–π stacking interactions. Single crystals of RP-H200 can be grown by dissolving IATH-1 into a solvent mixture of MeCN and dimethylformamide (DMF), followed by heating the solution at 90 °C for 12 h. See more details in Section S2.

In the single-crystal superstructure of RP-H200, each wing of IATH-1 engages (Figure 2b) in π–π stacking exclusively with another IATH-1, forming discrete π-dimers that are infinitely dispersed in a long-range order. The π–π stacking distances range (Figure 2b) from 3.38 to 3.55 Å, indicating the strong van der Waals interactions. It is noteworthy that the formation of such precisely discrete^47,51^ π-dimers is fundamentally important for studying the properties of π-aggregates in the solid state. Additionally, each wing of IATH-1 forms multiple hydrogen bonding interactions with another two IATH-1 molecules. The introduction of two hydrogen bonding motifs in each wing is essential for maintaining the noncoplanar conformation of IATH-1 during assembly. Nine IATH-1 molecules assemble (Figure 2c) into a secondary hexagonal unit through the formation of 12 [N–H···O] and 12 [O–H···N] hydrogen bonds, which exhibit distances ranging from 2.60 to 2.69 Å between nitrogen and oxygen atoms. Within this hexagonal unit, all IATH-1 molecules adopt the preferred noncoplanar conformation (Figure 1b), as the multiple hydrogen bonds between each pair of wings constrain the free rotation of individual wings along C–C bonds, thereby maintaining the noncoplanarity. Every two hexagonal units (in blue and magenta) can further interpenetrate (Figure 2d) with each other through the orchestration of π–π stacking and hydrogen bonding interactions. In total, 18 π-dimers are formed within the interpenetrated hexagonal unit. Additionally, eight strong [O–H···O] hydrogen bonds are formed, cross-linking the two interpenetrated hexagonal units as evidenced by the short hydrogen bonding distance (2.46 Å) between oxygen atoms.

**Figure 3:**
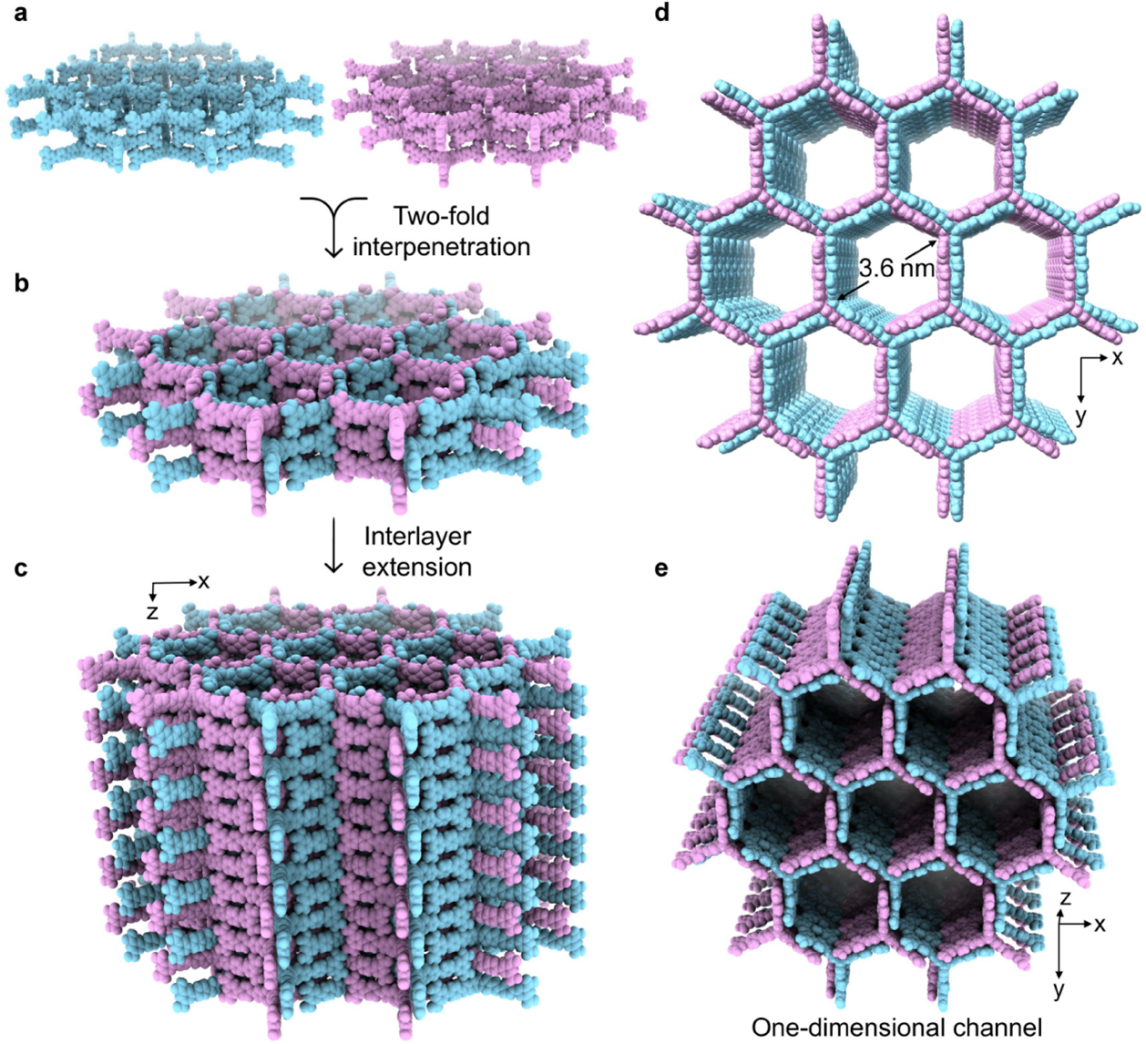
Single-crystal superstructures of the double-walled interpenetrated frameworks. (a–c) Two single-walled honeycomb-like frameworks, depicted in light blue and magenta, assembled (a) from IATH-1 molecules through [N–H···O] and [O–H···N] hydrogen bonding interactions, interpenetrates with each other to form (b) 2-fold interpenetrated frameworks, which further extend along the *z-*axis to form (c) the three-dimensional double-walled frameworks. (d, e) Top view (d) and side view (e) of the double-walled frameworks show one-dimensional hexagonal channels with a dimension of 3.6 nm.

The extension of the hexagonal unit shown in Figure 2c along the *x*–*y* plane, facilitated by the formation of [N–H···O] and [O–H···N] hydrogen bonds, leads (Figure 3a) to a layer of single-walled honeycomb-like framework. Within each layer, two single-walled frameworks (one in blue and one in magenta) interpenetrate with each other through π–π stacking and hydrogen-bonding interactions, resulting (Figures 3b and S1) in a 2-fold interpenetrated structure, namely, the double-walled framework. This structure further extends along the *z-*axis (Figure 3c) to form the three-dimensional framework, featuring (Figure S2) arrays of one-dimensional channels dominated by aromatic surfaces. Based on the single-crystal superstructure, the pore size of RP-H200 is determined (Figure 3d,e) to be 3.6 nm, which is the largest pore size among reported HOFs so far. See more details in Table S2.

### Stability and Porosity

**Figure 4:**
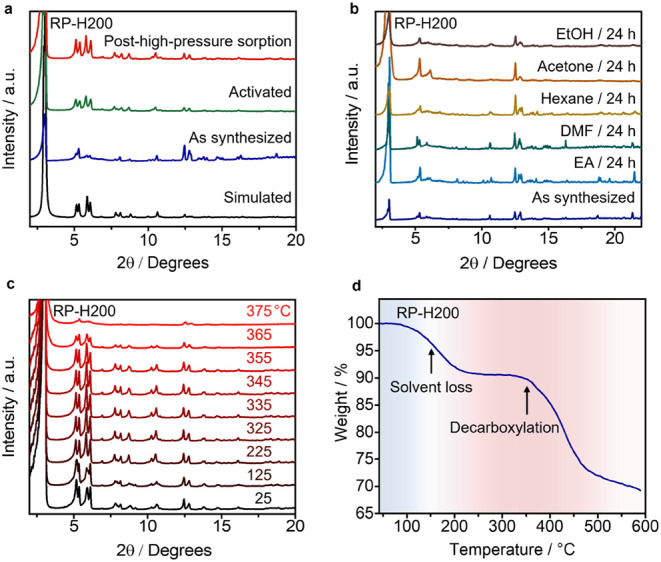
(a) PXRD patterns of as-synthesized, activated, and posthigh-pressure sorption RP-H200 samples match well with the simulated pattern of the single-crystal superstructures. The PXRD pattern of posthigh-pressure sorption corresponds to the sample after undergoing three rounds of methane and two rounds of hydrogen adsorption–desorption experiments at 100 bar. (b) PXRD Patterns of RP-H200 crystals before and after being immersed in various organic solvents for 24 h. EA, DMF, and EtOH represent ethyl acetate, dimethylformamide, and ethanol, respectively. (c) VT-PXRD Patterns of RP-H200 ranging from 25 to 375 °C. (d) TGA Curve of RP-H200 shows two steps of weight loss.

The multiple hydrogen bonding interactions, π–π stacking, and 2-fold interpenetration are expected to impart RP-H200 with high stability. Powder X-ray diffraction (PXRD) patterns of RP-H200 in its as-synthesized, activated, and post-high-pressure sorption states match well (Figure 4a) with the simulated pattern from single-crystal superstructures. To further validate this agreement, the refined cell parameters obtained from PXRD (Figure S6) were analyzed and show good consistency with those derived from single-crystal XRD data, confirming both phase purity and structural robustness upon solvent removal and high-pressure gas sorption/desorption. Additionally, RP-H200 displays good solvent stability. The PXRD patterns of RP-H200 remain (Figure 4b) consistent after immersing the crystals in commonly used organic solvents, such as DMF, ethanol, and acetone for 24 h. The framework also exhibits good stability under humid conditions, as evidenced by the retained crystallinity in the PXRD patterns (Figure S13).

Variable-temperature PXRD (VT-PXRD) and thermogravimetric analysis (TGA) were employed to evaluate the thermal stability of RP-H200. The VT-PXRD patterns (Figures 4c and S5) show almost no change upon heating up to 355 °C. TGA profiles reveal (Figure 4d) two weight loss steps: the first step corresponds to solvent release, while the second step beginning at ca. 350 °C is associated with the partial thermal decomposition of the frameworks. VT-PXRD and TGA results indicate that RP-H200 maintains good crystallinity and stability at elevated temperatures.

**Figure 5:**
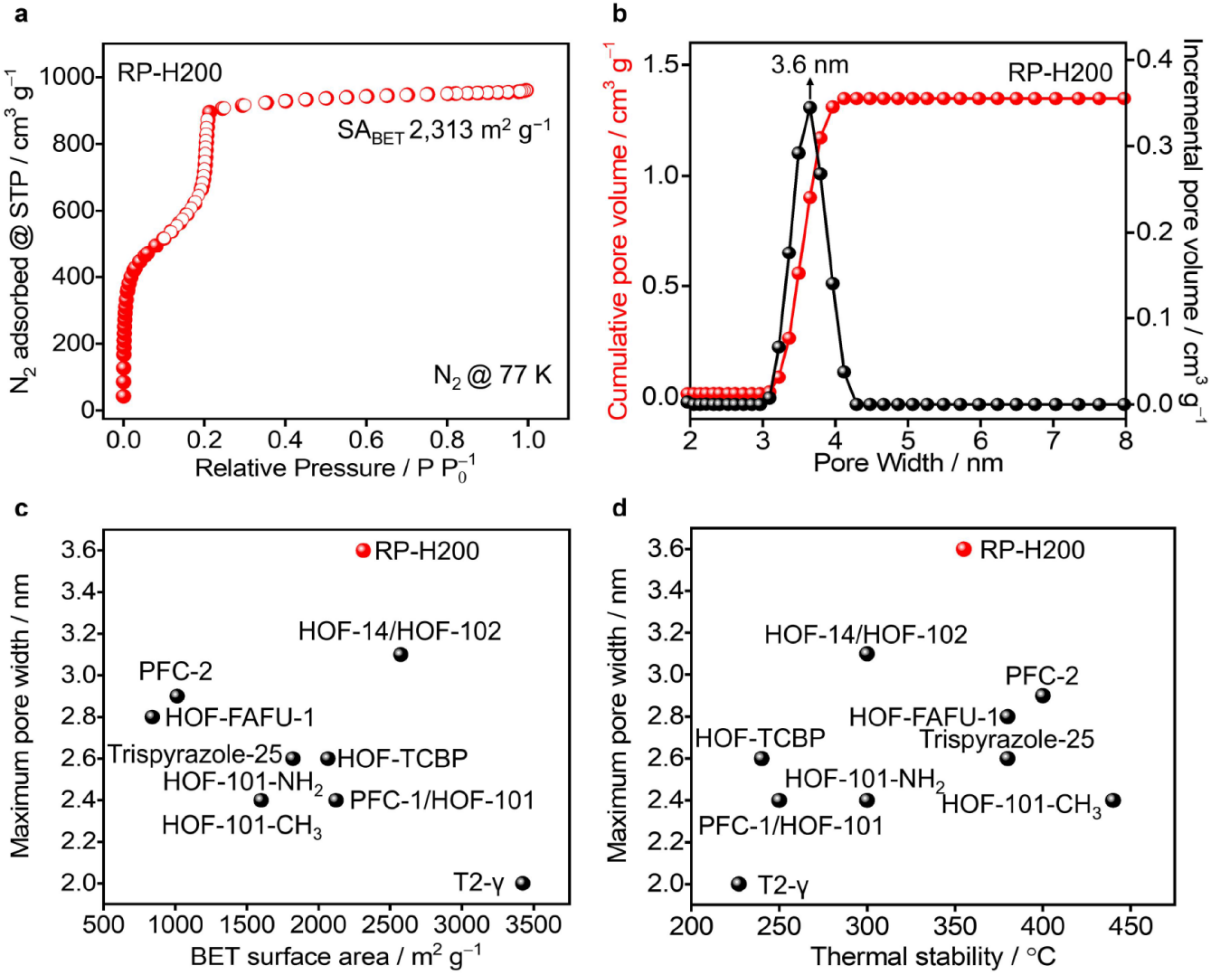
(a) The N_2_ adsorption (red filled circles) and desorption (red unfilled circles) isotherms for RP-H200 collected at 77 K. STP Denotes standard temperature and pressure, while SA_BET_ represents the BET surface area. (b) Pore size distribution of RP-H200, derived from the N_2_ isotherms at 77 K. (c, d) Trade-off between BET surface area and maximum pore width (c), and between thermal stability and maximum pore width (d), among all mesoporous HOFs with a pore width larger than 2 nm reported to date.

The porosity of RP-H200 was evaluated (Figure 5a) using N_2_ sorption isotherms at 77 K, which exhibits type IV sorption behavior, indicative of a mesoporous structure. This type IV sorption behavior is characterized by a steep uptake at low relative pressure (*P*/*P*_0_ < 0.01), followed by a noticeable plateau at higher *P*/*P*_0_. The gravimetric BET surface area, deduced from the N_2_ isotherms using (Figure S7) the updated Rouqueroí criteria in BETSI software^52^ is determined to be 2313 m^2^g^–1^. The mesoporous nature of RP-H200 is further supported by pore size distribution analysis derived from the N_2_ isotherms using the NLDFT method,^33^ revealing a pore size centered around 3.6 nm (Figure 5b), which is consistent with the value determined from single-crystal superstructures. The pore volume of RP-H200 is measured at 1.44 cm^3^ g^–1^, consistent with the theoretical value of 1.35 cm^3^ g^–1^ calculated from the single-crystal superstructure, and is one of the highest values among all reported HOFs (Table S2). More importantly, RP-H200 exhibits (Figure 5c) a coexistence of a high surface area and a large pore width, and presents (Figure 5d) a balance of high thermal stability and large pore width, both of which are important for its future applications.

### Clean Energy Gas Storage

**Figure 6:**
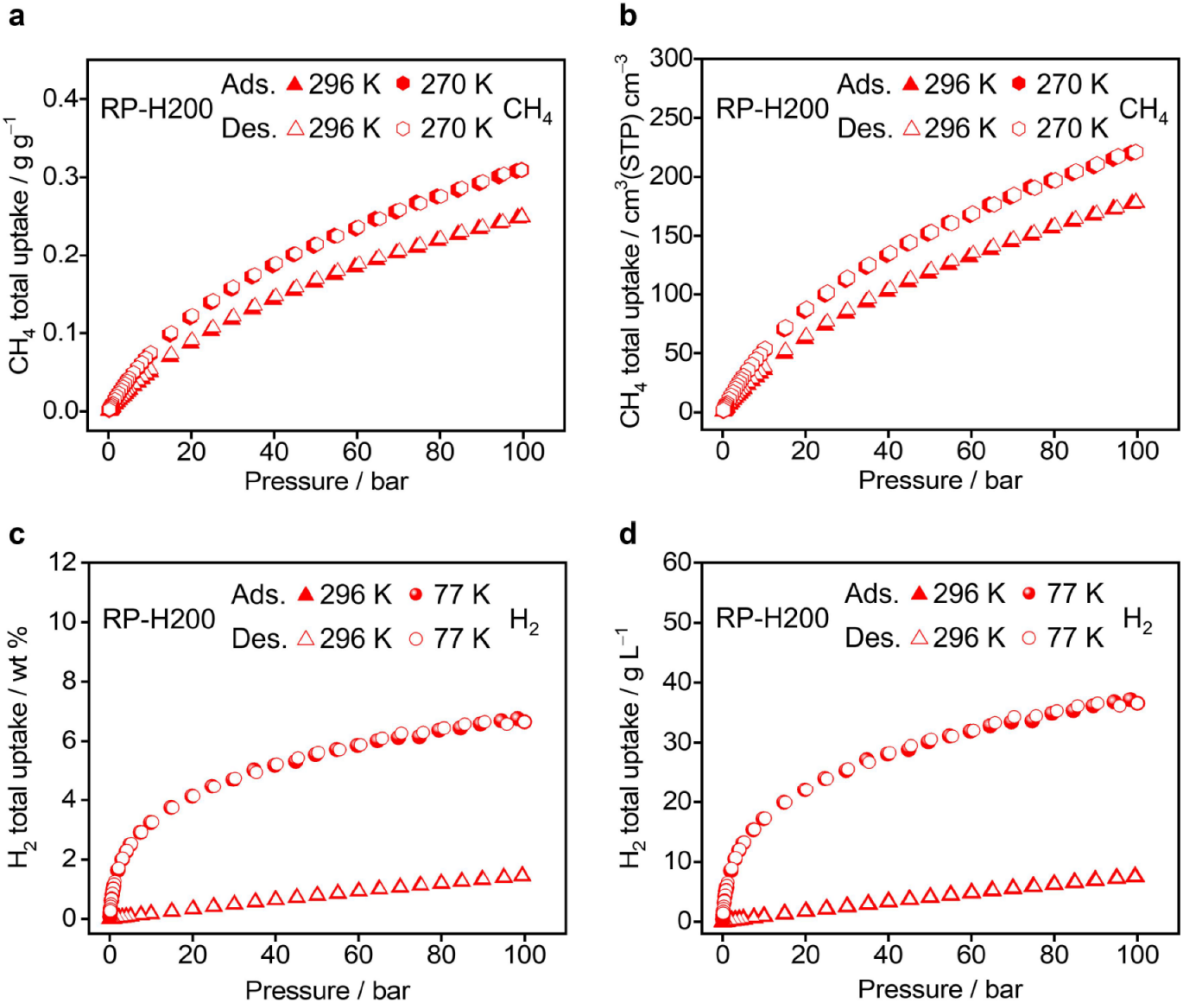
(a, b) Gravimetric (a) and volumetric (b) total methane uptakes in RP-H200 at 296 K (red triangles) and 270 K (red hexagons). (c, d) Gravimetric (c) and volumetric (d) total hydrogen uptakes in RP-H200 at 296 K (red triangles) and 77 K (red spheres). Adsorption data are represented by filled symbols, while desorption data are indicated by unfilled symbols.

The high porosity and aromatic pore chemistry^53^ of RP-H200 have inspired us to investigate its performance in storing clean energy gases, such as methane. As illustrated in Figures 6a,b and S8, RP-H200 exhibits a total gravimetric methane capacity of 0.31 g g^–1^ at 270 K/100 bar and 0.25 g g^–1^ at 296 K/100 bar, respectively. The total volumetric capacities for methane are determined to be 221 cm^3^ cm^–3^ at 270 K/100 bar, and178 cm^3^ cm^–3^ at 296 K/100 bar. The methane uptake capacity remains consistent after three adsorption–desorption cycles (Figure S12). Notably, the total gravimetric uptake of methane in RP-H200 at 296 K/100 bar is higher than that of many representative framework materials (see details in Table S3). The aromatic surfaces exposed toward the channels can introduce^48^ C–H−π interactions with methane, potentially contributing to its high uptake. The methane adsorption in RP-H200 correlates (Figure S9) with a moderate isosteric heat of adsorption (*Q*_st_) at around 12 kJ mol^–1^, which is similar to those of reported^31,54,55^ porous framework materials with high methane storage capacities.

Considering that the operating pressure for vehicles powered by methane is typically 5 bar, it is essential to assess the deliverable capacity, as gas adsorbed below this pressure is undeliverable. The deliverable capacities of methane in RP-H200 are determined to be 191 cm^3^ cm^–3^ (0.267 g g^–1^) and 158 cm^3^ cm^–3^ (0.221 g g^–1^) between pressures of 5 and 100 bar, measured at 270 and 296 K, respectively. Notably, the gravimetric deliverable capacity of RP-H200 at 270 K (0.267 g g^–1^) exceeds that of HKUST-1 (0.159 g g^–1^), while its volumetric deliverable capacity (191 cm^3^ m^–3^) is comparable to that of HKUST-1 (195 cm^3^ cm^–3^). In addition to its methane storage performance, RP-H200 also demonstrates potential for hydrogen storage. As shown in Figure 6c,d, RP-H200 shows a total hydrogen uptake of 6.7 wt % (36.6 g L^–1^) at 77 K/100 bar, and a total hydrogen uptake of 1.44 wt % (7.5 g L^–1^) at 296 K/100 bar, respectively. Additionally, RP-H200 shows relatively low water uptake (Figure S13), highlighting its potential^31,56^ for the storage of commercial gases that commonly include trace amount of water.

## Conclusions

In summary, we have developed a mesoporous double-walled HOF, RP-H200, which features the largest pore size—measuring 3.6 nm—among reported HOFs to date. The uniform distribution of precisely discrete π-dimers within RP-H200 enhances the utilization of molecular surfaces. RP-H200 exhibits a large surface area of 2313 m^2^ g^–1^ with a coexistence of high thermal stability. The framework possesses a high methane storage capacities of 0.31 g g^–1^ (221 cm^3^ cm^–3^) at 270 K/100 bar and 0.25 g g^–1^ (178 cm^3^ cm^–3^) at 296 K/100 bar, positioning it as a competitive candidate among reported framework materials. This research demonstrates a viable pathway for constructing robust mesoporous HOFs with diverse pore chemistries, paving the avenue for future applications.

## Supporting Information Available

Detailed experimental procedures, characterization data including single-crystal X-ray diffraction analyses, field emission scanning electron microscopy (FESEM) images, powder X-ray diffraction analyses, thermogravimetric analysis results, and sorption measurements (PDF)
