# Supplementary material for: Double-Walled Mesoporous Hydrogen-Bonded Organic Frameworks with High Methane Storage Capacity
Source: J Am Chem Soc. 2025 Apr 29;147(19):16412–9. doi: 10.1021/jacs.5c02705 (PMC12082628; doi:10.1021/jacs.5c02705)
Supplement: Supplementary file 1 — ja5c02705_si_001.pdf [file ja5c02705_si_001.pdf]

Supporting Information

Double-Walled Mesoporous Hydrogen-Bonded Organic Framework with High Methane Storage Capacity

Ruihua Zhang[1,2]*, Chun Tang[1,2]*, Shuliang Yang[2,3], Penghao Li[2,4], Han Han[1,2], Yong Wu[1,2], Guangcheng Wu[1,2], Xueze Zhao[1,2], Bai-Tong Liu[1,2], Sheng-Nan Lei[1], Bohan Tang[1], Enxu Liu[1], Yi-Kang Xing[1,2], Charlotte L. Stern[2], Christos D. Malliakas[2], J. Fraser Stoddart[1,2,5,6,7,8]◆

[1]*Department of Chemistry, The University of Hong Kong, Hong Kong SAR 999077, China*
[2]*Department of Chemistry, Northwestern University, 2145 Sheridan Road, Evanston, Illinois 60208, United States*
[3]*College of Energy, College of Chemistry and Chemical Engineering, Xiamen University, Xiamen, Fujian 361005, China.*
[4]*Department of Chemistry, The University of Mississippi, Mississippi 38677, United States*
[5]*Center for Regenerative Nanomedicine, Northwestern University, 303 East Superior Street, Chicago, Illinois 60611, United States*
[6]*Stoddart Institute of Molecular Science, Department of Chemistry, Zhejiang University, Hangzhou 310027, China*
[7]*ZJU-Hangzhou Global Scientific and Technological Innovation Center, Hangzhou 311215, China*
[8]*School of Chemistry, University of New South Wales, Sydney, New South Wales 2052, Australia*
*Correspondence to: ruihua@hku.hk, tangchem@hku.hk
◆*Deceased on December 30, 2024*

Table of Contents

1. Materials and General Methods 2
2. Singe-Crystal X-Ray Diffraction Analyses 2
3. Field Emission Scanning Electron Microscopy (FE-SEM) 5
4. Powder X-Ray Diffraction Analyses 6
5. Thermogravimetric Analyses 9
6. Sorption Measurements 9
7. References 21

1. Materials and General Methods

All reagents were purchased from commercial suppliers (AmBeed, Combi-Blocks, Sigma-Aldrich, or Fisher) and were used without further purification. Thin layer chromatography (TLC) was performed on silica gel 60 F254 (E. Merck). Normal-phase column chromatography (RediSep Rf Gold® Normal-Phase Silica) was conducted using CombiFlash® Automation Systems (Teledyne ISCO). Scanning electron microscopic (SEM) images were acquired using a Hitachi SU8030 FE-SEM at voltages of 10 kV, while energy-dispersive X-ray spectroscopy (EDS) elemental maps were collected at 15 kV.

2. Singe-Crystal X-Ray Diffraction Analyses

Crystallization procedure of RP-H200. IATH-1 was synthesized following the previously reported procedure[1]. IATH-1 (36 mg) was dissolved in DMF (3 mL), then the suspension was filtered through a 0.7 µm Whatman to remove particulates while the clear solution was added to a 6-dram borosilicate glass vial (22 mL) containing MeCN (3 mL). The tightly sealed vial was then put into a 90 °C oven for 12 h.

Data collection procedure of RP-H200. A suitable crystal was chosen and positioned on a MiTeGen holder in paratone oil on an XtaLAB Synergy R, DW system, and HyPix diffractometer. Throughout data collection, the crystal was kept at 225.15 K. Utilizing Olex2[2], the structure was solved with the SHELXT[3] structure solution program using Intrinsic Phasing and refined with the SHELXL[4] refinement package using Least Squares minimization.

Crystal Data for RP-H200. $C_{65}H_{38}N_6O_{12}$, (M =1095.01 g/mol): monoclinic, space group $C2/c$ (no. 15), a = 33.2612(9) Å, b = 60.174(3) Å, c = 14.1954(3) Å, β = 92.621(2)°, V = 28381.7(19) Å^3, Z = 8, T = 225.15 K, μ(Cu$K\alpha$) = 0.297 mm^{-1}, D_{calc} = 0.513 g cm^{-3}, 115838 reflections measured ($5.146 \leq 2\Theta \leq 127.38$), 23346 unique ($R_{int}$ = 0.1055, R_{sigma} = 0.0556) which were used in all calculations. The final R_1 was 0.1258 ($I > 2\sigma$(I)) and wR_2 was 0.4116 (all data).

Refinement Details of RP-H200. A minor twin fraction of 1.43% was identified and modeled using the twin matrix (-0.466 -0.489 0.057 -1.603 0.466 -0.172 0 0 -1) during the refinement process. The enhanced rigid-bond restraint (SHELX keyword RIGU)[5] was applied to disordered components. One of the disordered oxygen atoms was refined with isotropic displacement parameters.

Solvent Treatment Details of RP-H200. The solvent masking procedure implemented in Olex2 was used to remove the electronic contribution of solvent molecules from the refinement. As the exact solvent content is unknown, only the atoms used in the refinement model are reported in the formula here. Total solvent accessible volume / cell = 19624 Å^3 [69.1%] Total electron count / cell = 3856.

The crystallographic data of RP-H200 is available from the Cambridge Crystallographic Data Centre (CCDC) via www.ccdc.cam.ac.uk/data_request/cif. The CCDC number is 2416539.

Table S1. Crystal data and structure refinement for RP-H200

Empirical formula	$C_{65}H_{38}N_6O_{12}$
Formula weight	1095.01
Temperature / K	225.01(10)
Crystal system	monoclinic
Space group	*C*2/c
a / Å, b / Å, c / Å	33.2612(9), 60.174(3), 14.1954(3)
α/°, β/°, γ/°	90, 92.621(2), 90
Volume / Å^3	28381.7(19)
Z	8
ρ_{calc} / g cm^{-3}	0.513

μ / mm^{-1}	0.297
F(000)	4528.0
Crystal size / mm^3	0.232 × 0.076 × 0.054
2Θ range for data collection	5.146 to 127.38°
Index ranges	$-35 \le h \le 38$, $-69 \le k \le 57$, $-16 \le l \le 16$
Reflections collected	23346
Independent reflections	23346 [R(int) = 0.1055, R(sigma) = 0.0556]
Data/restraints/parameters	23346/0/739
Goodness-of-fit on F^2	1.093
Final R indexes [I>2σ (I)]	R_1 = 0.1258, wR_2 = 0.3800
Final R indexes [all data]	R_1 = 0.1544, wR_2 = 0.4116
Largest diff. peak/hole / e Å^{-3}	0.50/–0.42

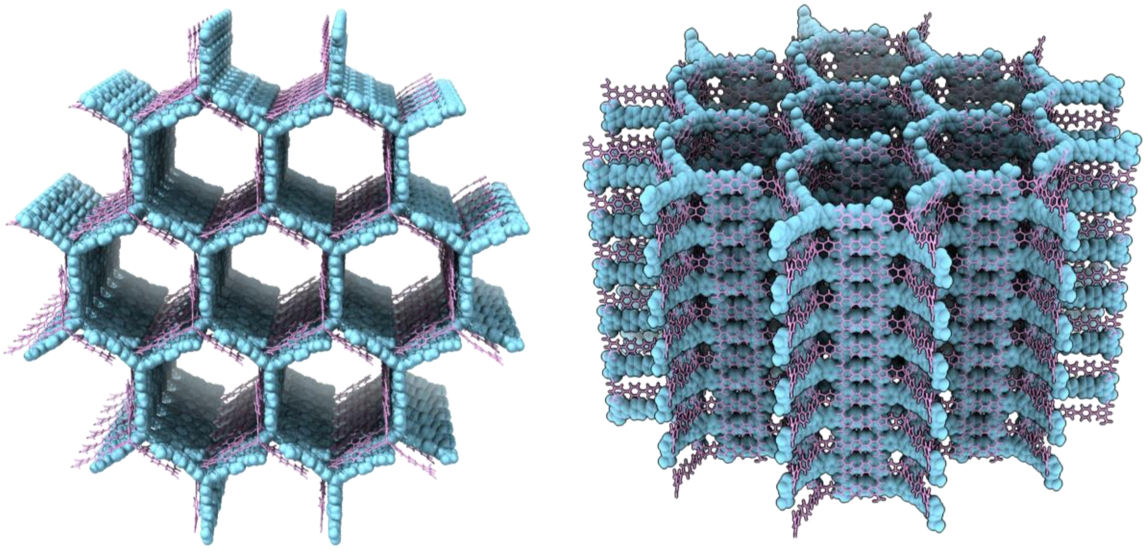

Figure S1. Different views of the two-fold interpenetrated frameworks in RP-H200: one single-walled framework displayed in blue (sphere mode) and the other single-walled framework in magenta (stick mode), highlighting the aromatic surfaces predominantly exposed to the channel.

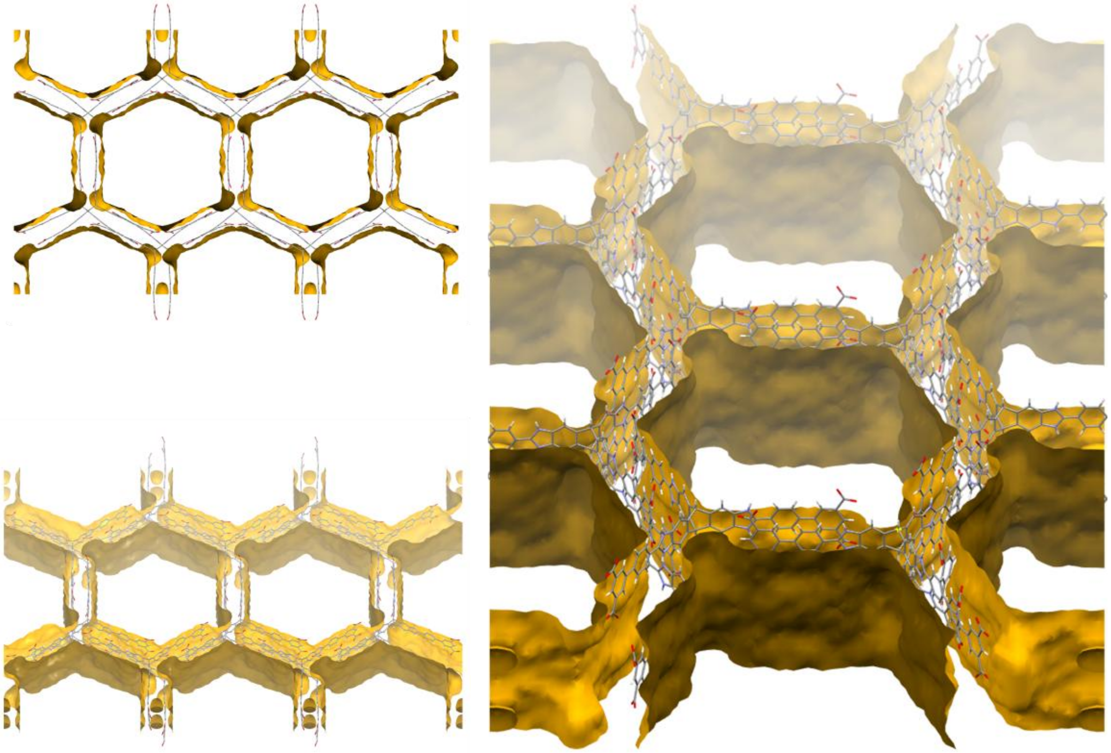

Figure S2. One-dimensional channel structure of the void space in RP-H200.

3. Field Emission Scanning Electron Microscopy (FE-SEM)

FE-SEM characterizations were conducted using a Hitachi S-4800 at the EPIC/NUANCE facility at Northwestern University. The dried RP-H200 crystalline samples were affixed to carbon conductive tape. After that, a thin layer of approximately 9 nm of OsO_4 was evaporated to the crystal surface using a Denton Desk III TSC Sputter Coater to enhance conductivity.

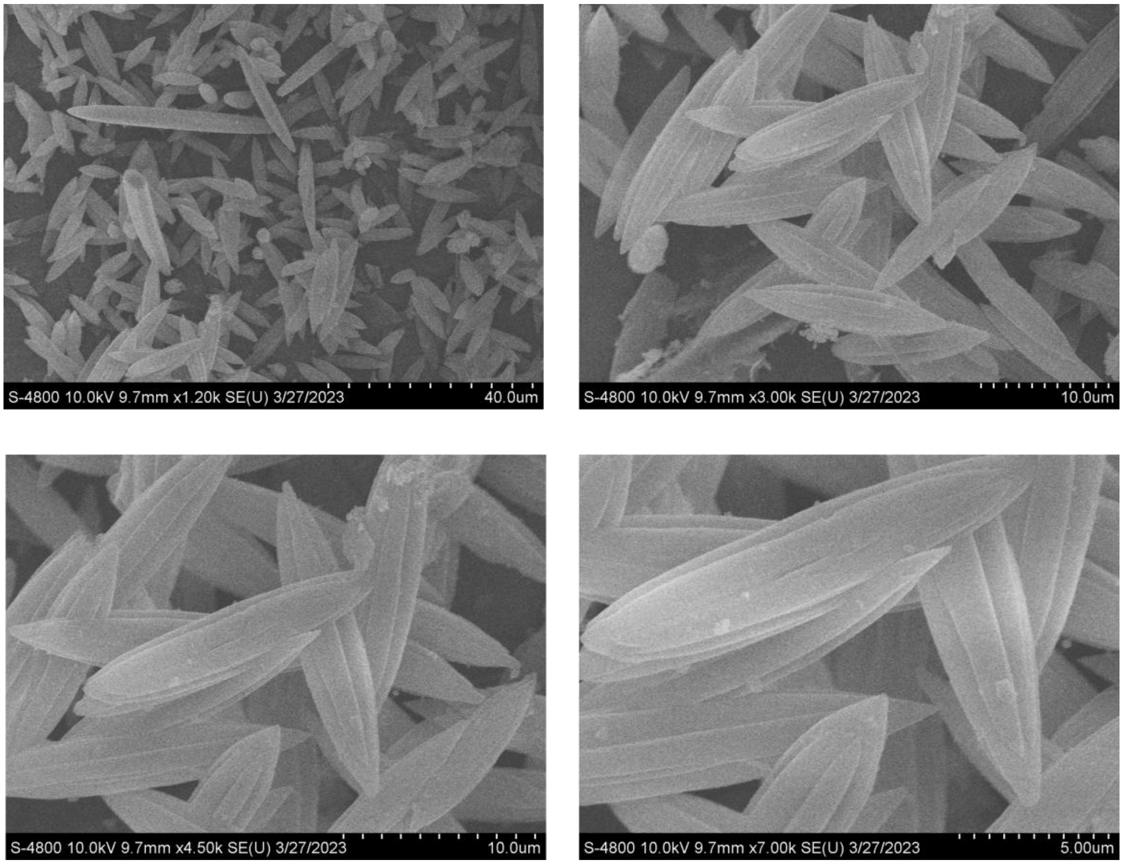

Figure S3. SEM images of RP-H200 crystals at different magnifications.

4. Powder X-Ray Diffraction Analyses

PXRD Data were collected using a STOE-STADI-P powder diffractometer equipped with an asymmetrically curved Germanium monochromator (CuKα1 radiation, λ = 1.54056 Å) and one-dimensional silicon strip detector (MYTHEN2 1K from DECTRIS) at room temperature. The line-focused Cu X-ray tube was operated at 40 kV and 40 mA. Single crystals of HOF samples were packed in a 3-mm metallic mask and taped with two layers of polyimide tape. Intensity data from the two thetas of 2 to 20 degrees were collected over 10 mins. Before the measurements, the instrument was calibrated against a NIST Silicon standard (640d). The simulated PXRD patterns were generated based on the single crystal data using the Mercury software.

Variable temperature PXRD (VT-PXRD) data were acquired on a STOE-STADI-MP powder diffractometer equipped with an asymmetrically curved Germanium monochromator (Cu$K\alpha1$ radiation, λ = 1.54056 Å) and one-dimensional silicon strip detector (MYTHEN2 1K from DECTRIS) at room temperature. The line-focused Cu X-ray tube was operated at 40 kV and 40 mA. The vacuum-dried HOF crystals were packed in a 0.8 mm borosilicate capillary and inserted into the furnace. Temperature stability is typically 0.1 °C. Intensity data from the two thetas of 2 to 20 degrees were collected over 10 mins. The instrument was calibrated against a NIST Silicon standard (640d) before the measurement.

Note: A small discrepancy is observed between the PXRD patterns in Figure 4a and 4c, which is most likely due to differences in sample preparation methods and sample quantities.

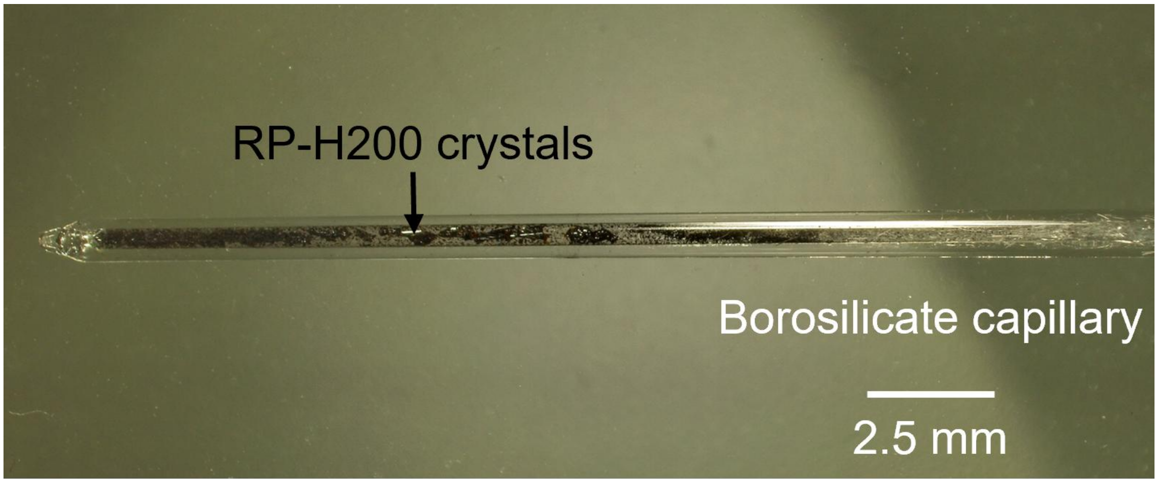

Figure S4. RP-H200 crystalline samples were packed in borosilicate capillaries for VT-PXRD characterization.

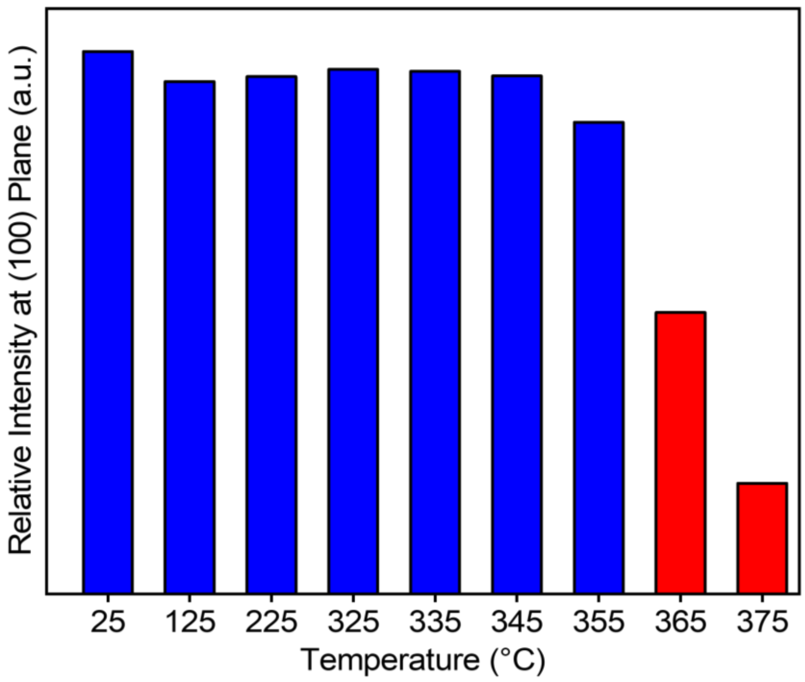

Figure S5. The relative intensity of PXRD peaks at (110) planes of **RP-H200** under different temperatures.

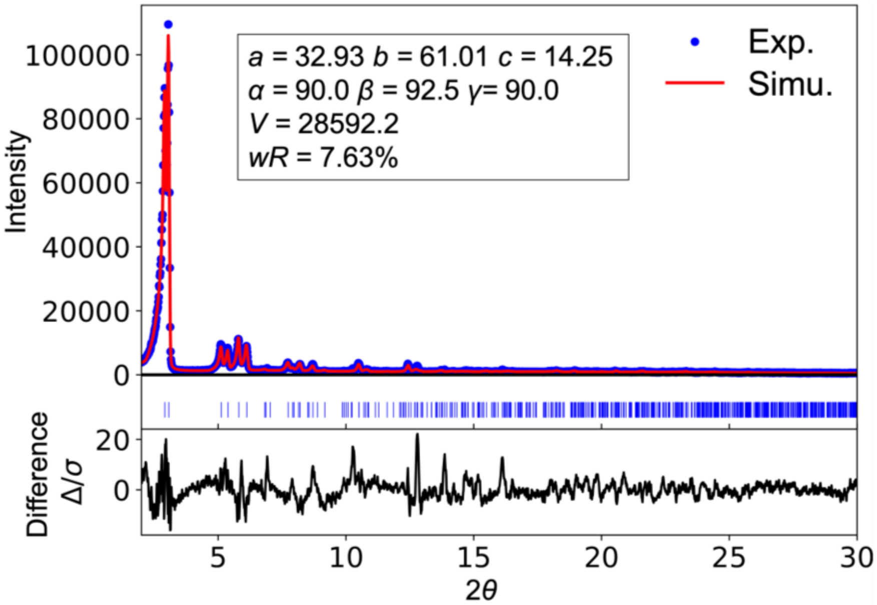

Figure S6. Experimental PXRD pattern of the post-high-pressure sorption RP-H200 sample (blue) alongside the Rietveld refined pattern (red), obtained using the GSAS-II software. The difference between the experimental and simulated data is shown in black at the bottom, and the refined unit cell parameters are marked with a black square.

5. Thermogravimetric Analyses

Thermogravimetric analyses were conducted in a Netzsch STA 449 F3 Jupiter Simultaneous Thermal Analysis (STA) instrument. Single crystals of HOF materials (~10 mg) were placed in a pre-weighed alumina crucible (~200 mg) and weighed three times. The thermogravimetric analysis was performed under ultra-high purity helium gas (50 mL/min). To account for the buoyancy effect of helium gas, the empty crucible was measured under the same conditions as the samples, and the results were used for correction. The temperature was increased at a rate of 10 °C/min and gases were transferred to a GC/MS through a heated (250 °C) transfer line. An Agilent Technologies 7890A GC system, equipped with a non-polar capillary column (Agilent J&B HP-5 packed with (5%-phenyl)-methylpolysiloxane) coupled to a 5975 MSD spectrometer, was used for analyzing the gases released from the samples. Gas injection was triggered every 10 min during the heating cycle, and 0.25 mL of gas was sampled from the gases released by the sample and carrier gas (He). The mass spectra were collected in the range of 10–400 u. The thermo-balance of the STA was verified for performance up to 1000 °C using a certified sample of calcium oxalate monohydrate (European Pharmacopoeia Reference Standard).

6. Sorption Measurements

The as-synthesized crystalline sample of RP-H200 was activated by supercritical CO_2 (sc-CO_2) in a Tousimis Samdri PVT-30 critical point dryer. Prior to the sc-CO_2 drying, the residual solvent from the as-synthesized samples was removed using a pipette. The remaining crystals were then washed with DMF and immersed in DMF for 2 days. The DMF solvent was refreshed every 12 hours. During each refreshment, the DMF was discarded before 4 mL of fresh DMF was added into the vial. After 2 days, most of the DMF was removed. The remaining crystals were soaked in MeCN for another 3 days to exchange the remaining DMF thoroughly. During the solvent exchange process, MeCN was refreshed every 12 h. After another 2 days, we removed most of the MeCN, and transferred the RP-H200 crystals into a glass container for sc-CO_2 activation. The crystalline sample after activation was quickly transferred in open air to a sorption tube of ASAP 2020 plus and then degassed at 90 °C for 2 h and 140 °C for 12 h.

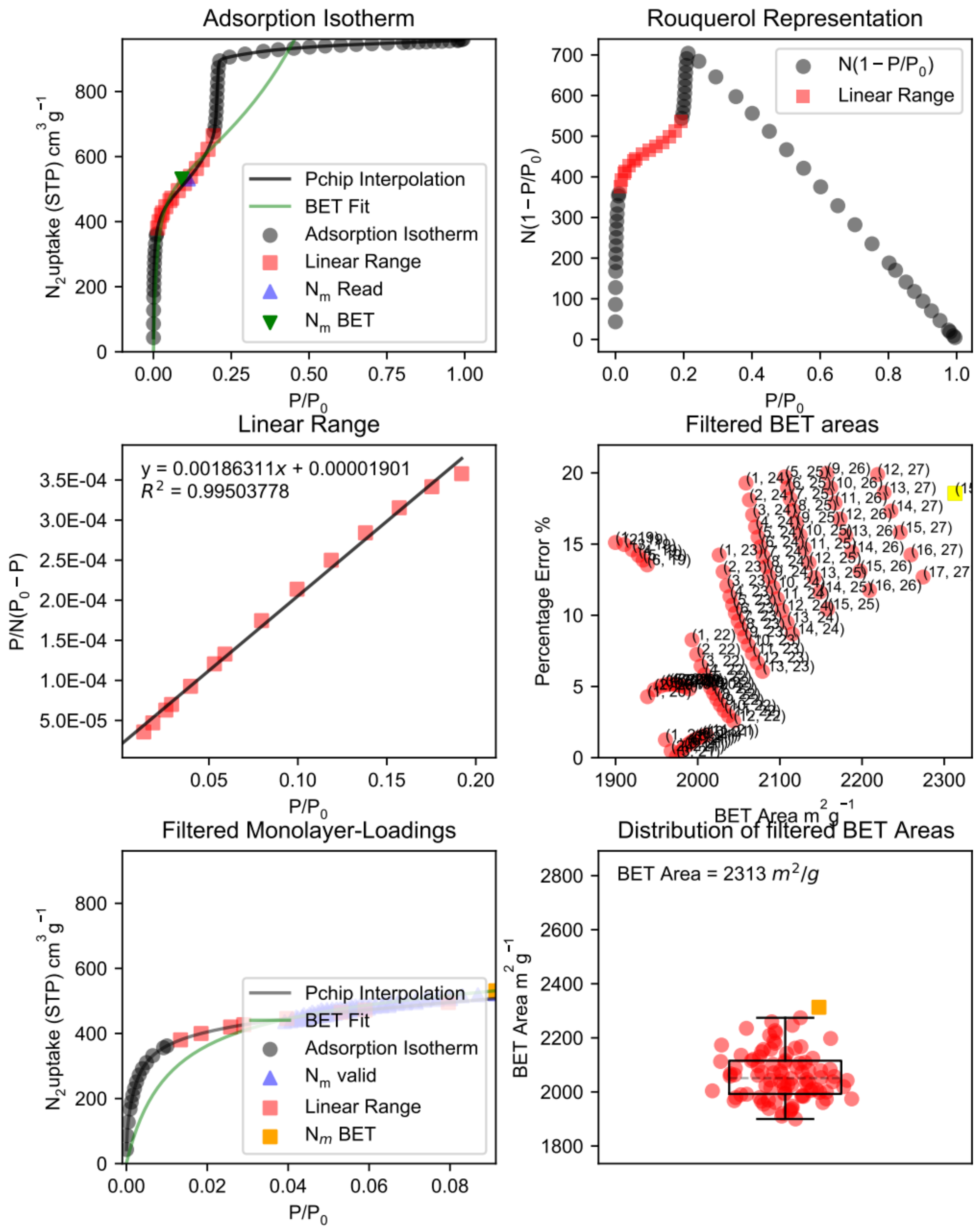

Figure S7. BET Surface area of RP-H200 analyzed by BETSI from the N_2 adsorption isotherms at 77 K.

Nitrogen adsorption-desorption isotherm. Helium and nitrogen used for sorption measurements were ultra-high purity (UHP) grade, 99.999%. N_2 adsorption-desorption isotherms of RP-H200 were collected on an ASAP 2020 plus (Micromeritics) at 77 K. The updated Rouqueroĺ criteria implemented software BETSI[6] was used to calculate the Brunauer–Emmett–Teller (BET) surface areas from the N_2 sorption isotherms to improve reproducibility. The pore size distributions were derived from N_2 isotherms at 77 K using Non-Local Density Functional Theory (NLDFT) calculations, employing a cylindrical pore model.

High-pressure methane and hydrogen sorption measurements.

After activating the RP-H200 sample with supercritical carbon dioxide (sc-CO_2) and degassing at 25 °C for 2 hours, followed by 90 °C for 2 hours and finally at 140 °C for 12 hours in the degas station of an ASAP 2020 Plus, and finished the subsequent ambient-pressure nitrogen isotherms measurement on ASAP 2020 plus. The samples were then quickly transferred to a pre-cleaned, dried, and pre-weighed 2-mL microcell and sealed for weighing, which was subsequently attached to the iSorb HP1. After that, RP-H200 crystals were degassed using iSorb HP1 at 90 °C for 2 h under a dynamic vacuum.

High-pressure sorptions of methane and hydrogen were characterized using an iSorb HP1 (Anton Paar) instrument controlled by a computer. The helium and hydrogen used for sorption measurements were ultra-high purity (UHP) grade, 99.999%. Methane was sourced as UHP grade at 99.99%.

The instrument operates at 45 °C for the manifold and between –198 to 500 °C for the sample cell, with pressure ranging from vacuum up to 100 bar. This apparatus measures the excess adsorption amount of the adsorbate based on a volumetric method. A controlled quantity of adsorbate (hydrogen or methane) is dosed from the manifold (at 45 °C) into the sample cell, which is maintained at the analysis temperature. For measurements at 77 K,

temperature control is achieved using a liquid nitrogen system that automatically replenishes liquid nitrogen to maintain a constant level. This ensures that both the sample cell and the connecting tubing to the manifold remain at a stable temperature throughout the experiment, which is critical for accurate measurements. Additionally, room temperature is kept stable during the entire measurement process.

For analyses at 296 K and 270 K, a circulator bath containing a 50/50 volume mixture of ethylene glycol and distilled water is used as the bath fluid. Throughout the measurements, temperature and pressure in both the manifold and sample cell are continuously monitored. The difference between the actual analysis temperature and the set temperature remains below 0.02 K for the manifold and less than 0.5 K for the samples.

Errors in weighing samples (averaged from more than five measurements) and pressure readings consistently remained below 0.5%, resulting in overall measurement errors of less than 0.05 wt%, which is negligible. Potential sources of error include the selection of an appropriate equation of state (EOS) for non-ideal gas conditions and temperature approximations in the connecting tubing between the sample cell and manifold. The mBWR-Jacobsen EOS was utilized for processing helium data, while the mBWR EOS was used for hydrogen and methane data reduction *[NIST Standard Reference Database 23: NIST Reference Fluid Thermodynamic and Transport Properties Database]*. It is noteworthy that temperature approximation errors in the connecting tubing are more pronounced at 77 K due to the significant temperature difference between the sample cell and the manifold in this region. A filler rod is inserted into the connecting tubing between the manifold and sample cell, to provide a sharper temperature interface between the manifold and the sample cell. In this situation, dividing the connection region into two zones—a cold zone and a warm zone—can get a more accurate void volume correction. To estimate and minimize measurement errors, all sample measurements were repeated using the same cell without any sample present at identical pressure and temperature

conditions, yielding an "adsorption" of less than 0.05 mmol g^{-1}. During data reduction, blank subtraction was performed on each sample measurement to account for any adsorption attributed to the empty cell. The void volumes of the empty cell were determined through helium expansion tests conducted at both manifold temperature (45 °C) and sample analysis temperature after each blank measurement. After high-pressure gas sorption experiments, void volumes of cells containing samples were re-evaluated under both manifold temperature (45 °C) and analysis temperature conditions. These void volumes were essential for calculating adsorbed amounts of adsorbate.

It is important to note that only excess adsorption amounts (N_{ex})—the additional gas adsorbed due to the presence of adsorbents—are directly measurable experimentally. In contrast, absolute adsorption (N_{abs}) is defined as the sum of excess adsorption (N_{ex}) plus the volume of gas that fills the pore space at gas-phase density. Absolute adsorption can be calculated using:

$$N_{abs} = N_{ex} + V_p \cdot \rho_{bulk} (P, T)$$

The pore volume of the adsorbent materials, denoted as V_p (cm^3 g^{-1}), can be derived from N_2 isotherms at a relative pressure of P/P_0 = 0.95 / 77 K, whilst ρ_{bulk} refers to the bulk density of the adsorbate at the specific temperature and pressure during measurement. For this analysis, we utilized the experimentally measured bulk density data obtained from NIST.[7].

The gravimetric capacity of methane, expressed in g g^{-1}, is calculated based on the formula: g g^{-1} = (mass of CH_4) / (mass of HOFs).The gravimetric capacity of hydrogen, expressed as wt%, is calculated using the formula wt% = (mass of H_2) / (mass of HOFs + mass of H_2) × 100%. For the calculations of volumetric capacity, the crystallographic density was used.

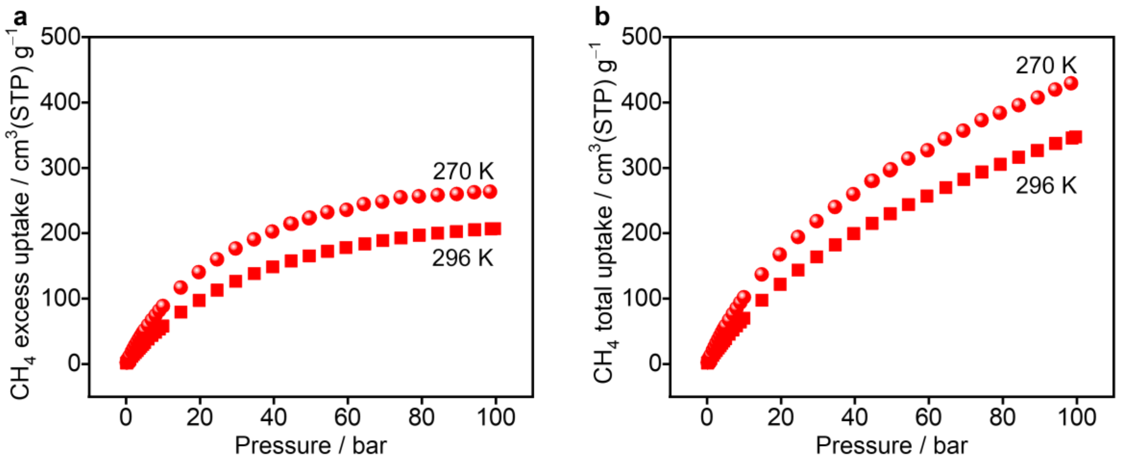

Figure S8. Experimental high-pressure CH_4 excess (**a**) and total (**b**) uptakes of RP-H200 at 296 and 270 K, respectively.

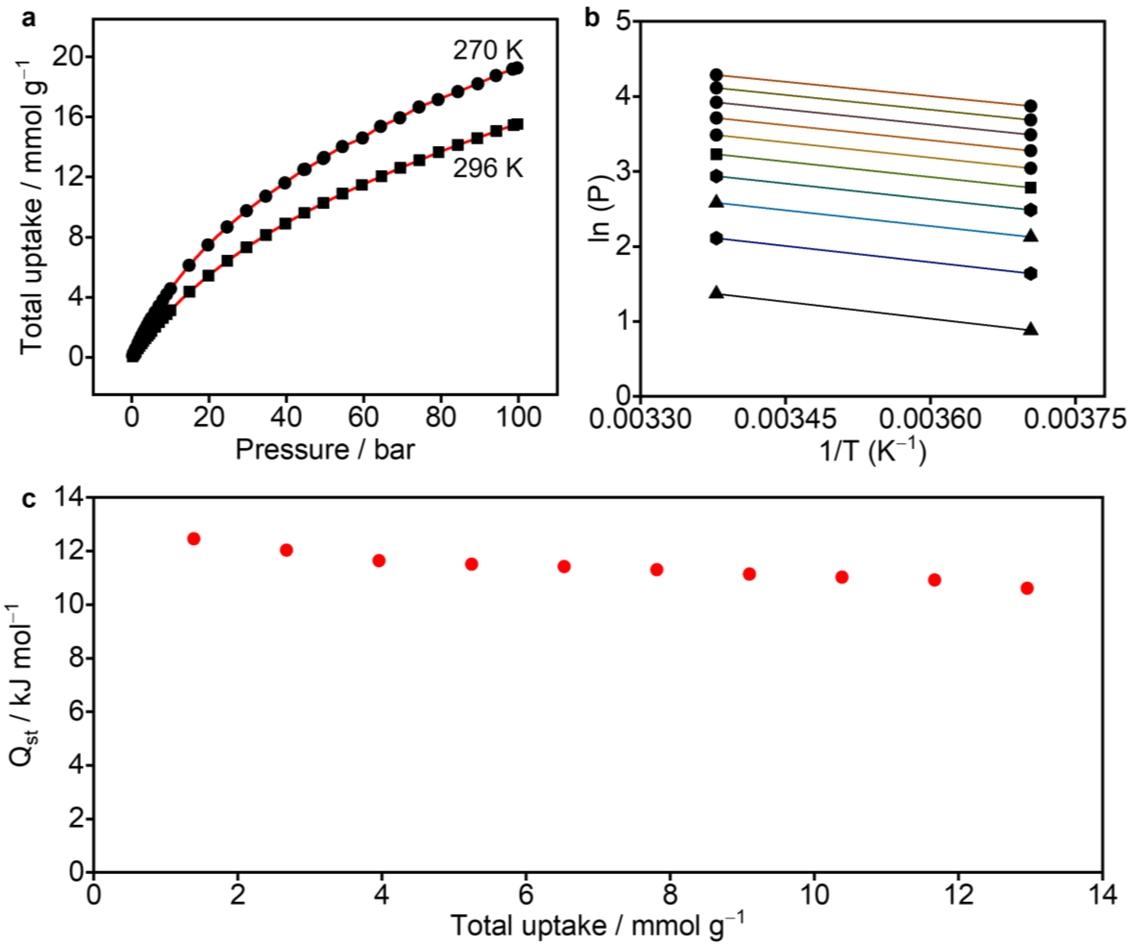

Figure S9. **a**, The CH_4 adsorption isotherms for RP-H200 at 270 and 296 K. **b**, Corresponding Van't Hoff Plot derived from (**a**). **c**, The isosteric heats of adsorption (Q_{st}) of RP-H200 for CH_4.

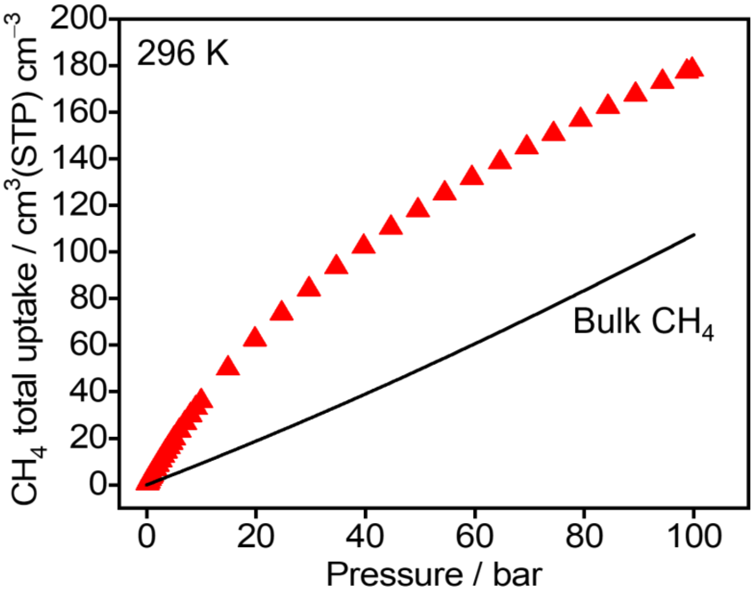

Figure S10. The CH_4 total uptake isotherm for RP-H200 in comparison with bulk CH_4 density at 296 K.

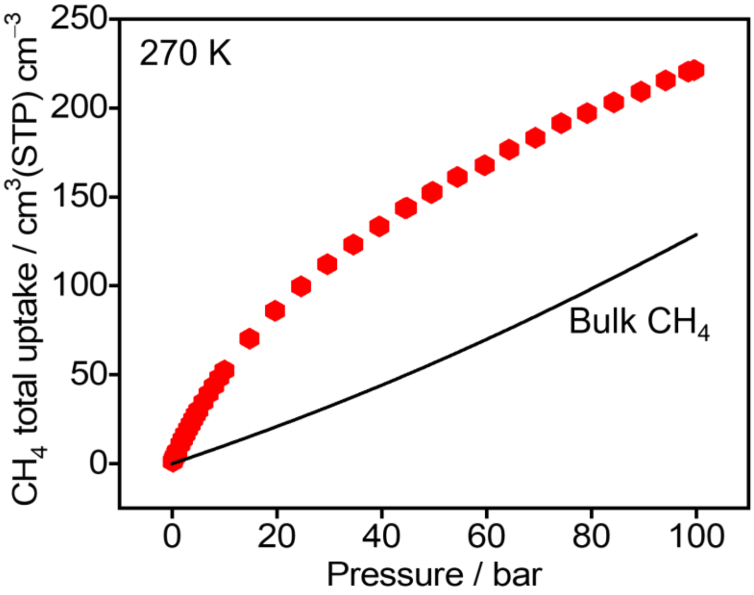

Figure S11. The CH_4 total uptake isotherm for RP-H200 in comparison with bulk CH_4 density at 270 K.

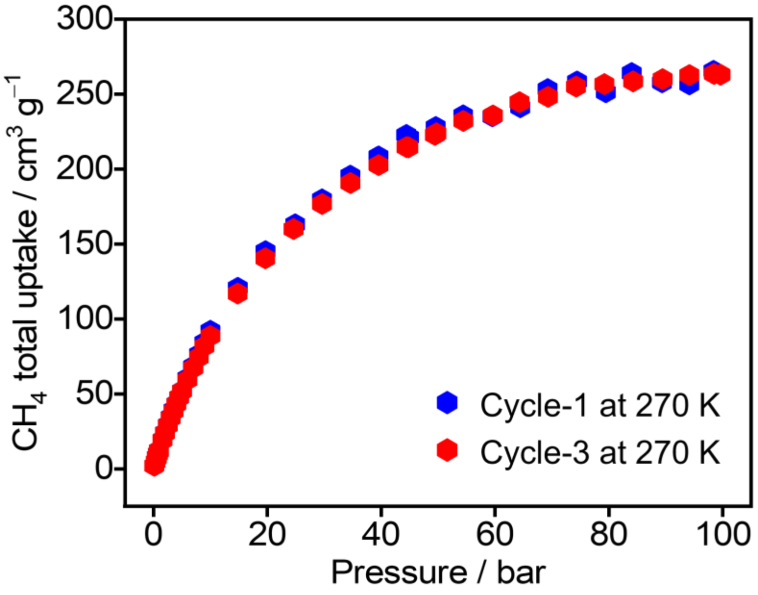

Figure S12. Multiple cycles of high-pressure CH_4 sorption for RP-H200. Blue and red hexagons correspond to the first cycle and the third cycle of CH_4 sorption measurement at 270 K, respectively.

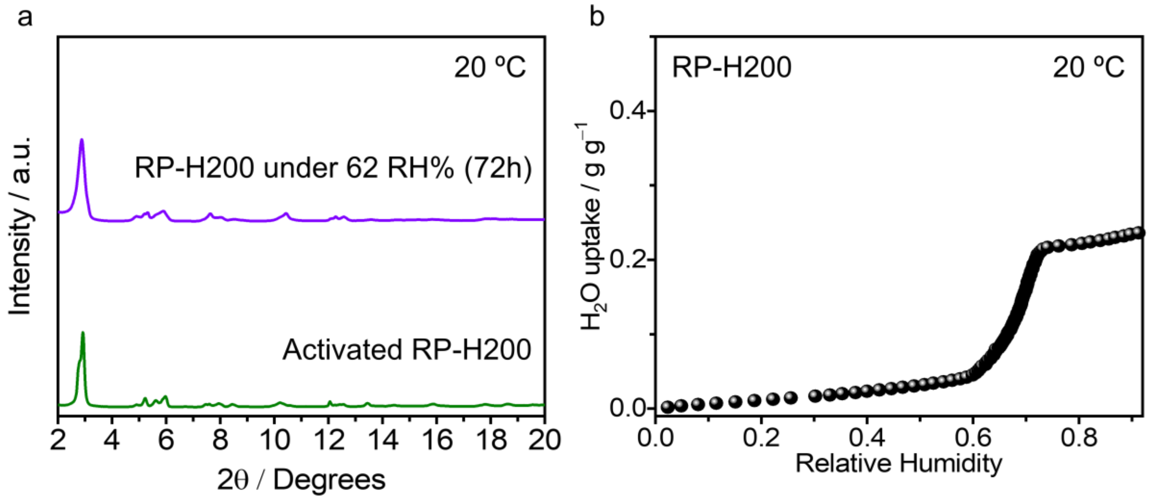

Figure S13. (a) PXRD patterns of activated RP-H200 before and after exposed to moisture with a relative humidity of approximately 60 % at 20 ºC for 72 h. (b) Water vapor adsorption isotherm for RP-H200 at 20 ºC.

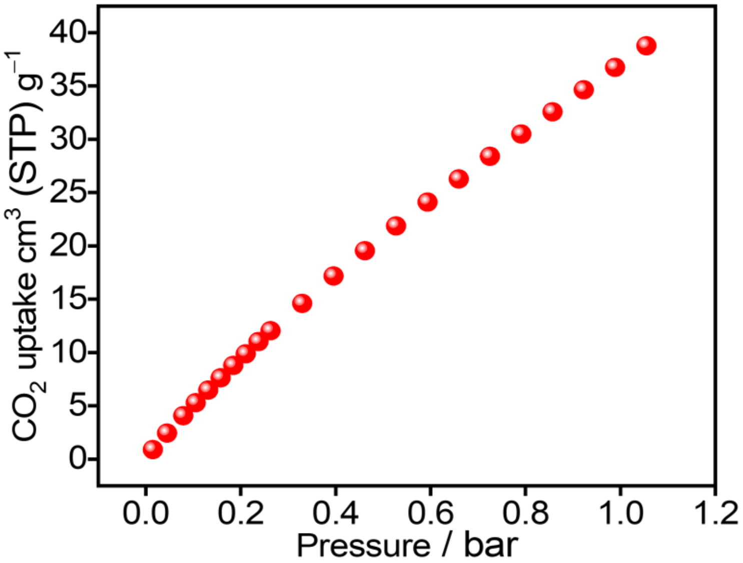

Figure S14. The CO_2 adsorption isotherm for RP-H200 at 293 K. STP stands standard temperature and pressure.

Table S2. The porosity and thermal stability for HOFs from reported literature[8]

Materials	GSA ($m^2\ g^{-1}$)	VSA[a] ($m^2\ g^{-1}$)	Density[b] ($g\ cm^{-3}$)	Pore width[c] (nm)	Pore volume[e] ($cm^3\ g^{-1}$)	Thermal stability (°C)	Ref.
PFC-1/HOF-101	2122	1572	0.741	1.8 × 2.4	0.95	250	[9]
HOF-101-NH_2	1600	1272	0.795	1.8 × 2.4	/	300	[10]
HOF-101-CH_3	1600	1272	0.795	1.8 × 2.4	/	440	[10]
HOF-14/HOF-102	2573	1724	0.670	2.4 × 3.1	1.36	300	[11-12]
Trispyrazole-25	1821	1471	0.808	2.6	0.72[f]	380	[13]
CPDBC-1a	1548	1098	0.709	1.3	0.64[f]	360	[14]
HOF-TCBP	2066	1428	0.691	1.8 × 2.6	0.83	240	[15]
TTBI	2796	2167	0.755	1.45	1.02	/	[16]
T2-γ	3425	1428	0.417	2.0	1.57	227	[17]
TH5-A	3284	1228	0.374	1.9	1.66[f]	120	[18]

ZJU-HOF-10	1169	655	0.560	1.6	/	360	[19]
PETHOF-1	1150	371	0.323	1.1[d]	0.48	340	[20]
PETHOF-2	1140	945	0.829	1.1[d]	0.46	380	[20]
CBPHAT-1a	1288	1095	0.850	1.45	0.55	339	[21]
tet-$[2\cdot(TP)_2]_n$	1473	1208	0.820	1.3	/	225	[22]
Tcpb/H_3BTB	1095	1061	0.969	1.85[d]	0.42	180	[23]
HOF-BTB	955	908	0.951	1.66	0.40	300	[24]
ZJU-HOF-1	1465	1269	0.866	0.7	0.60	200	[25]
HOF-76a	1121	1123	1.002	0.7	0.40	300	[26]
ABTPA-2	1183	745	0.630	1.8	0.62	300	[27]
ThiaHAT-1a	1394	1175	0.843	1.6	0.64	305	[28]
HOF-20a	1323	1172	0.886	1.3	0.57	400	[29]
HOF-5a	1101	1051	0.955	0.4 × 0.7	0.44	400	[30]
Trispyrazole-1	1159	1227	1.059	1.65	0.48[f]	360	[31]
PFC-2	1014	529	0.522	2.9	/	400	[32]
HOF-FAFU-1	840	650	0.774	1.6 × 2.8	0.27	380	[33]
RP-H100	2383	1573	0.660	1.7	1.11	375	[1]
RP-H101	3526	1855	0.526	1.8	1.35	375	[1]
**RP-H200**	**2313**	**1187**	**0.513**	**3.6**	**1.44**	**355**	**This work**

[a]VSA is calculated based on GSA and crystallographic density.

[b]Densities derived based on single-crystal superstructure.

[c]Pore width determined from single crystal superstructure.

[d]Pore width derived from N_2 isotherms at 77 K.

[e]Pore volume determined from N_2 isotherms at 77 K.

[f]Theoritical pore volume, calculated from density and void fraction.

Table S3. Methane storage capacities for selected HOFs, COFs, POPs and MOFs

Type	Materials	*ρ*[a]	Total uptake[d]		Deliverable uptake[k]		Q_{st}	Ref.
		g cm^{-3}	cm^3 cm^{-3}	g g^{-1}	cm^3 cm^{-3}	g g^{-1}	kJ mol^{-1}	
HOFs	**RP-H200**	**0.51**	**178 (221)**	**0.25 (0.31)**	**158 (191)**	**0.22 (0.27)**	**12.0**	**This work**
	ZJU-HOF-5a	0.64	192 (232)	0.22 (0.26)	159 (187)	0.18 (0.21)	21.5	[34]
	SOF-1a	1.22	39[e]	0.02[e]	20[l]	0.01[l]	20.8	[35]
	TACM-HOF	1.12	73[i] (60)	0.05[i] (0.04)	N/A	N/A	21.4	[36]
	SOF-7a	1.31	50[f]	0.03[f]	28[m]	0.02[m]	N/A	[37]
COFs	COF-1	0.98[b]	89[g]	0.07[g]	67[n]	0.05[n]	17.1	[38]
	COF-5	0.58[b]	154[g]	0.19[g]	127[n]	0.16[n]	8.9	[38]
	COF-8	0.71[b]	150[g]	0.15[g]	130[n]	0.13[n]	11.9	[38]
	COF-102	0.41[c]	191[g]	0.33[g]	165[n]	0.29[n]	9.2	[38]
	COF-320	0.73	176[g]	0.17[g]	151[n]	0.15[n]	19.2	[39]
	3D-TFB-COF-Me	0.42	249[j] (286)	0.423[j] (0.487)	223[p] (248)	0.38[p] (0.42)	11.0–14.0	[40]
	3D-TFB-COF-Et	0.44	264[j] (296)	0.429[j] (0.482)	237[p] (256)	0.39[p] (0.42)	10.5–11.5	[40]
POPs	PAF-1	0.33[c]	187	0.41	156[l]	0.34[l]	14.0	[41-42]
	PPN-1	0.13[c]	36[g]	0.20[g]	32[n]	0.18[n]	18.4	[43]
	PPN-4	0.28[c]	155[h]	0.39[h]	136[o]	0.34[o]	N/A	[44]
	PPN-13	0.22[b]	77	0.25	68	0.22	13.9	[45]
	KPOP-1	0.20[b]	128[g]	0.45[g]	115[n]	0.40[n]	16.6	[46]
	KPOP-2	0.19[b]	133[g]	0.52[g]	121[n]	0.47[n]	16.0	[46]
	KPOP-3	0.26[b]	97[g]	0.26[g]	81[n]	0.22[n]	19.9	[46]
	COP-150	0.34[b]	55	0.12	48	0.10	N/A	[47]
	SHCP-3-Cl	0.36[b]	153	0.30	121	0.24	16.3	[48]

**MOFs**	Ni-MOF-74	1.21	270	0.16	162 (135)	0.10 (0.08)	21.4	[49-50]
	HKUST-1	0.83	277	0.24	207 (195)	0.18 (0.17)	17.0	[49-50]

[a]Density derived based on single-crystal superstructure.

[b]Bulk density of material.

[c]Density derived from simulated crystal structure.

[d–h]Total methane uptake at room temperature (or 270 K) and [d]100 bar, [e]16 bar, [f]20 bar/273 K. [g]80 bar, [h]55 bar.

[i–j]Total methane uptake at room temperature (or 273 K) and [i]20 bar, [j]100 bar.

[k–o]The *P*–5 bar deliverable uptake of CH_4 at room temperature (or 270 K), *P* =[k]100 bar, [l]16 bar, [m]20 bar, [n]80 bar, [o]55 bar.

[p]The *P*–5 bar deliverable uptake of CH_4 at room temperature (or 273 K) and 100 bar.

Table S4. Methane storage capacities for RP-H200 and commercially viable materials

Type	Materials	ρ	Total uptake		Pressure	Temperature	Ref.
		g cm^{-3}	cm^3 cm^{-3}	g g^{-1}	bar	ºC	
**HOF**	**RP-H200**	**0.513**	**96**	**0.134**	**35**	**22.85**	**This work**
			**125**	**0.175**		**−3.15**	
**Activated Carbon**	Norit AC	N/A	N/A	0.069	35	26.85	[51]
	SRD-21	N/A	N/A	0.107	35	30	[52]
	CAQF	0.583	95	0.117	35	30	[52-53]
	AX-21	0.49	153	0.225	35	25	[49,54]
**Zeolite**	Zeolite NaX	1.43	106	0.053	35.5	25	[55]
	Zeolite 5A	1.48	104	0.050	35.5	5	[55]

Table S5. Hydrogen storage capacities for selected HOFs and MOFs

Types	Materials	GSA ($m^2 g^{-1}$)	ρ ($g cm^{-3}$)	VSA ($m^2 cm^{-3}$)	Total capacity[a] (wt%)[b]	Total capacity[a] ($g L^{-1}$)[c]	Ref.
MOFs	MOF-5	3510	0.605	2070	8.0	52.6	[51]
	IRMOF-20	4070	0.510	2080	9.3	52.7	[51]
	NU-1101	4340	0.459	1992	9.5	48.7	[52]
	NU-1102	3720	0.403	1499	9.9	45.3	[52]
	NU-1103	6245	0.298	1861	13.0	44.9	[52]
	NU-125	3230	0.578	1867	8.3	52.1	[53]
	NU-1000	2200	0.571	1256	8.0	49.9	[53]
	PCN-250	1780	0.896	1595	5.4	51.2	[53]
	UiO-68-Ant	3030	0.607	1839	7.6	50.2	[53]
	HKUST-1	1980	0.879	1740	5.4	49.7	[53]
	$Zn(BDC)_2$ (DABCO)	2020	0.873	1763	4.9	45.3	[53]
	NOTT-112	3440	0.446	1534	8.8	43.0	[53]
	CYCU-3-A1	2450	0.447	1095	8.3	40.5	[53]
	UiO-67	2360	0.688	1624	5.9	43.2	[53]
	Cu-MOF-74	1270	1.323	1680	3.2	43.4	[53]
	rht-MOF-7	1950	0.789	1539	4.9	40.8	[53]
	SNU-70	4944	0.411	2030	10.7	49.2	[54]
	NU-100	6050	0.290	1755	14.2	48.0	[54]
	UMCM-9	5039	0.370	1860	11.6	48.5	[54]
	NU-1501-A1	7310	0.283	2060	14.5	47.9	[55]
	NU-1501-Fe	7140	0.299	2130	13.7	47.3	[55]
	NU-1500-A1	3560	0.498	1770	8.6	46.8	[55]
HOFs	ZJU-HOF-5a	3102	0.637	1976	6.4	43.2	[56]
	RP-H100	2383	0.660	1573	6.5	45.8	[1]
	RP-H101	3526	0.526	1855	9.7	56.5	[1]
	**RP-H200**	**2313**	**0.513**	**1187**	**6.7**	**36.8**	**This work**

[a]Total H_2 uptake capacity is determined by measuring the uptake at 77 K/100 bar. [b]Gravimetric H_2 capacity (wt%) is calculated based on wt% = (mass of H_2) / (mass of H_2 + mass of adsorbents) × 100%. [c]Volumetric capacity ($g L^{-1}$) is calculated based on crystallographic density.

7. References

(1) Zhang, R.; Daglar, H.; Tang, C.; Li, P.; Feng, L.; Han, H.; Wu, G.; Limketkai, B. N.; Wu, Y.; Yang, S.; Chen, A. X. Y.; Stern, C. L.; Malliakas, C. D.; Snurr, R. Q.; Stoddart, J. F. Balancing Volumetric and Gravimetric Capacity for Hydrogen in Supramolecular Crystals. *Nat. Chem.* **2024,** *16*, 1982–1988.

(2) Dolomanov, O. V.; Bourhis, L. J.; Gildea, R. J.; Howard, J. A.; Puschmann, H. OLEX2: A Complete Structure Solution, Refinement and Analysis Program. *J. Appl. Crystallogr.* **2009,** *42*, 339–341.

(3) Sheldrick, G. SHELXT – Integrated Space-Group and Crystal-Structure Determination. *Acta Crystallogr. A* **2015,** *71*, 3–8.

(4) Sheldrick, G. A Short History of SHELX. *Acta Crystallogr. A* **2008,** *64*, 112–122.

(5) Thorn, A.; Dittrich, B.; Sheldrick, G. M. Enhanced Rigid-Bond Restraints. *Acta Crystallogr. Sect. A: Found. Crystallogr.* **2012,** *68*, 448–451.

(6) Osterrieth, J. W. M.; Rampersad, J.; Madden, D.; Rampal, N.; Skoric, L.; Connolly, B.; Allendorf, M. D.; Stavila, V.; Snider, J. L.; Ameloot, R.; Marreiros, J.; Ania, C.; Azevedo, D.; Vilarrasa-Garcia, E.; Santos, B. F.; Bu, X.-H.; Chang, Z.; Bunzen, H.; Champness, N. R.; Griffin, S. L.; Chen, B.; Lin, R.-B.; Coasne, B.; Cohen, S.; Moreton, J. C.; Colón, Y. J.; Chen, L.; Clowes, R.; Coudert, F.-X.; Cui, Y.; Hou, B.; D'Alessandro, D. M.; Doheny, P. W.; Dincă, M.; Sun, C.; Doonan, C.; Huxley, M. T.; Evans, J. D.; Falcaro, P.; Ricco, R.; Farha, O.; Idrees, K. B.; Islamoglu, T.; Feng, P.; Yang, H.; Forgan, R. S.; Bara, D.; Furukawa, S.; Sanchez, E.; Gascon, J.; Telalović, S.; Ghosh, S. K.; Mukherjee, S.; Hill, M. R.; Sadiq, M. M.; Horcajada, P.; Salcedo-Abraira, P.; Kaneko, K.; Kukobat, R.; Kenvin, J.; Keskin, S.; Kitagawa, S.; Otake, K.-i.; Lively, R. P.; DeWitt, S. J. A.; Llewellyn, P.; Lotsch, B. V.; Emmerling, S. T.; Pütz, A. M.; Martí-Gastaldo, C.; Padial, N. M.; García-Martínez, J.; Linares, N.; Maspoch, D.; Suárez del Pino, J. A.; Moghadam, P.; Oktavian, R.; Morris, R. E.; Wheatley, P. S.; Navarro, J.; Petit, C.; Danaci, D.; Rosseinsky, M. J.; Katsoulidis, A. P.; Schröder, M.; Han, X.; Yang, S.; Serre, C.; Mouchaham, G.; Sholl, D. S.; Thyagarajan, R.; Siderius, D.; Snurr, R. Q.; Goncalves, R. B.; Telfer, S.; Lee, S. J.; Ting, V. P.; Rowlandson, J. L.; Uemura, T.; Iiyuka, T.; van der Veen, M. A.; Rega, D.; Van Speybroeck, V.; Rogge, S. M. J.; Lamaire, A.; Walton, K. S.; Bingel, L. W.; Wuttke, S.; Andreo, J.; Yaghi, O.; Zhang, B.; Yavuz, C. T.; Nguyen, T. S.; Zamora, F.; Montoro, C.; Zhou, H.; Kirchon, A.; Fairen-Jimenez, D. How Reproducible Are Surface Areas Calculated from the BET Equation? *Adv. Mater.* **2022,** *34*, 2201502.

(7) McCarty, R. D.; Arp, V. D., A New Wide Range Equation of State for Helium. In

Advances in Cryogenic Engineering: Part A & B, Springer: 1990; pp 1465–1475.
(8) Song, X.; Wang, Y.; Wang, C.; Wang, D.; Zhuang, G.; Kirlikovali, K. O.; Li, P.; Farha, O. K. Design Rules of Hydrogen-Bonded Organic Frameworks with High Chemical and Thermal Stabilities. *J. Am. Chem. Soc.* **2022,** *144*, 10663–10687.
(9) Yin, Q.; Zhao, P.; Sa, R.-J.; Chen, G.-C.; Lü, J.; Liu, T.-F.; Cao, R. An Ultra-Robust and Crystalline Redeemable Hydrogen-Bonded Organic Framework for Synergistic Chemo-Photodynamic Therapy. *Angew. Chem. Int. Ed.* **2018,** *57*, 7691–7696.
(10) Wang, Y.; Ma, K.; Bai, J.; Xu, T.; Han, W.; Wang, C.; Chen, Z.; Kirlikovali, K. O.; Li, P.; Xiao, J.; Farha, O. K. Chemically Engineered Porous Molecular Coatings as Reactive Oxygen Species Generators and Reservoirs for Long-Lasting Self-Cleaning Textiles. *Angew. Chem. Int. Ed.* **2022,** *61*, e202115956.
(11) Wang, B.; Lv, X.-L.; Lv, J.; Ma, L.; Lin, R.-B.; Cui, H.; Zhang, J.; Zhang, Z.; Xiang, S.; Chen, B. A Novel Mesoporous Hydrogen-Bonded Organic Framework with High Porosity and Stability. *Chem. Commun.* **2020,** *56*, 66–69.
(12) Ma, K.; Li, P.; Xin, J. H.; Chen, Y.; Chen, Z.; Goswami, S.; Liu, X.; Kato, S.; Chen, H.; Zhang, X.; Bai, J.; Wasson, M. C.; Maldonado, R. R.; Snurr, R. Q.; Farha, O. K. Ultrastable Mesoporous Hydrogen-Bonded Organic Framework-Based Fiber Composites toward Mustard Gas Detoxification. *Cell Rep. Phys. Sci.* **2020,** *1*, 100024.
(13) Hashim, M. I.; Le, H. T. M.; Chen, T.-H.; Chen, Y.-S.; Daugulis, O.; Hsu, C.-W.; Jacobson, A. J.; Kaveevivitchai, W.; Liang, X.; Makarenko, T.; Miljanić, O. Š.; Popovs, I.; Tran, H. V.; Wang, X.; Wu, C.-H.; Wu, J. I. Dissecting Porosity in Molecular Crystals: Influence of Geometry, Hydrogen Bonding, and [π···π] Stacking on the Solid-State Packing of Fluorinated Aromatics. *J. Am. Chem. Soc.* **2018,** *140*, 6014–6026.
(14) Suzuki, Y.; Tohnai, N.; Saeki, A.; Hisaki, I. Hydrogen-Bonded Organic Frameworks of Twisted Polycyclic Aromatic Hydrocarbon. *Chem. Commun.* **2020,** *56*, 13369–13372.
(15) Hu, F.; Liu, C.; Wu, M.; Pang, J.; Jiang, F.; Yuan, D.; Hong, M. An Ultrastable and Easily Regenerated Hydrogen-Bonded Organic Molecular Framework with Permanent Porosity. *Angew. Chem. Int. Ed.* **2017,** *56*, 2101–2104.
(16) Mastalerz, M.; Oppel, I. M. Rational Construction of an Extrinsic Porous Molecular Crystal with an Extraordinary High Specific Surface Area. *Angew. Chem. Int. Ed.* **2012,** *51*, 5252–5255.
(17) Pulido, A.; Chen, L.; Kaczorowski, T.; Holden, D.; Little, M. A.; Chong, S. Y.; Slater, B. J.; McMahon, D. P.; Bonillo, B.; Stackhouse, C. J.; Stephenson, A.; Kane, C. M.; Clowes, R.; Hasell, T.; Cooper, A. I.; Day, G. M. Functional Materials Discovery Using Energy–

Structure–Function Maps. *Nature* **2017,** *543*, 657–664.
(18) Shields, C. E.; Wang, X.; Fellowes, T.; Clowes, R.; Chen, L.; Day, G. M.; Slater, A. G.; Ward, J. W.; Little, M. A.; Cooper, A. I. Experimental Confirmation of a Predicted Porous Hydrogen-Bonded Organic Framework. *Angew. Chem. Int. Ed.* **2023,** *62*, e202303167.
(19) Wang, J.-X.; Gu, X.-W.; Lin, Y.-X.; Li, B.; Qian, G. A Novel Hydrogen-Bonded Organic Framework with Highly Permanent Porosity for Boosting Ethane/Ethylene Separation. *ACS Mater. Lett.* **2021,** *3*, 497–503.
(20) Li, P.; Li, P.; Ryder, M. R.; Liu, Z.; Stern, C. L.; Farha, O. K.; Stoddart, J. F. Interpenetration Isomerism in Triptycene-Based Hydrogen-Bonded Organic Frameworks. *Angew. Chem.* **2019,** *131*, 1678–1683.
(21) Hisaki, I.; Suzuki, Y.; Gomez, E.; Cohen, B.; Tohnai, N.; Douhal, A. Docking Strategy to Construct Thermostable, Single-Crystalline, Hydrogen-Bonded Organic Framework with High Surface Area. *Angew. Chem. Int. Ed.* **2018,** *57*, 12650–12655.
(22) Morshedi, M.; Thomas, M.; Tarzia, A.; Doonan, C. J.; White, N. G. Supramolecular Anion Recognition in Water: Synthesis of Hydrogen-Bonded Supramolecular Frameworks. *Chem. Sci.* **2017,** *8*, 3019–3025.
(23) Zentner, C. A.; Lai, H. W. H.; Greenfield, J. T.; Wiscons, R. A.; Zeller, M.; Campana, C. F.; Talu, O.; FitzGerald, S. A.; Rowsell, J. L. C. High Surface Area and Z′ in a Thermally Stable 8-Fold Polycatenated Hydrogen-Bonded Framework. *Chem. Commun.* **2015,** *51*, 11642–11645.
(24) Yoon, T.-U.; Baek, S. B.; Kim, D.; Kim, E.-J.; Lee, W.-G.; Singh, B. K.; Lah, M. S.; Bae, Y.-S.; Kim, K. S. Efficient Separation of C2 Hydrocarbons in a Permanently Porous Hydrogen-Bonded Organic Framework. *Chem. Commun.* **2018,** *54*, 9360–9363.
(25) Zhang, X.; Wang, J. X.; Li, L.; Pei, J.; Krishna, R.; Wu, H.; Zhou, W.; Qian, G.; Chen, B.; Li, B. A Rod-Packing Hydrogen-Bonded Organic Framework with Suitable Pore Confinement for Benchmark Ethane/Ethylene Separation. *Angew. Chem. Int. Ed.* **2021,** *60*, 10304–10310.
(26) Zhang, X.; Li, L.; Wang, J.-X.; Wen, H.-M.; Krishna, R.; Wu, H.; Zhou, W.; Chen, Z.-N.; Li, B.; Qian, G.; Chen, B. Selective Ethane/Ethylene Separation in a Robust Microporous Hydrogen-Bonded Organic Framework. *J. Am. Chem. Soc.* **2020,** *142*, 633–640.
(27) Cui, P.; Svensson Grape, E.; Spackman, P. R.; Wu, Y.; Clowes, R.; Day, G. M.; Inge, A. K.; Little, M. A.; Cooper, A. I. An Expandable Hydrogen-Bonded Organic Framework

Characterized by Three-Dimensional Electron Diffraction. *J. Am. Chem. Soc.* **2020,** *142*, 12743–12750.
(28) Suzuki, Y.; Gutiérrez, M.; Tanaka, S.; Gomez, E.; Tohnai, N.; Yasuda, N.; Matubayasi, N.; Douhal, A.; Hisaki, I. Construction of Isostructural Hydrogen-Bonded Organic Frameworks: Limitations and Possibilities of Pore Expansion. *Chem. Sci.* **2021,** *12*, 9607–9618.
(29) Wang, B.; He, R.; Xie, L.-H.; Lin, Z.-J.; Zhang, X.; Wang, J.; Huang, H.; Zhang, Z.; Schanze, K. S.; Zhang, J.; Xiang, S.; Chen, B. Microporous Hydrogen-Bonded Organic Framework for Highly Efficient Turn-Up Fluorescent Sensing of Aniline. *J. Am. Chem. Soc.* **2020,** *142*, 12478–12485.
(30) Wang, H.; Li, B.; Wu, H.; Hu, T.-L.; Yao, Z.; Zhou, W.; Xiang, S.; Chen, B. A Flexible Microporous Hydrogen-Bonded Organic Framework for Gas Sorption and Separation. *J. Am. Chem. Soc.* **2015,** *137*, 9963–9970.
(31) Chen, T.-H.; Popov, I.; Kaveevivitchai, W.; Chuang, Y.-C.; Chen, Y.-S.; Daugulis, O.; Jacobson, A. J.; Miljanić, O. Š. Thermally Robust and Porous Noncovalent Organic Framework with High Affinity for Fluorocarbons and CFCs. *Nat. Commun.* **2014,** *5*, 5131.
(32) Yin, Q.; Li, Y.-L.; Li, L.; Lü, J.; Liu, T.-F.; Cao, R. Novel Hierarchical Meso-Microporous Hydrogen-Bonded Organic Framework for Selective Separation of Acetylene and Ethylene Versus Methane. *ACS Appl. Mater. Interfaces* **2019,** *11*, 17823–17827.
(33) Lin, Z.-J.; Qin, J.-Y.; Zhan, X.-P.; Wu, K.; Cao, G.-J.; Chen, B. Robust Mesoporous Functional Hydrogen-Bonded Organic Framework for Hypochlorite Detection. *ACS Appl. Mater. Interfaces* **2022,** *14*, 21098–21105.
(34) Wang, J. X.; Zhang, X.; Jiang, C.; Zhang, T. F.; Pei, J.; Zhou, W.; Yildirim, T.; Chen, B.; Qian, G.; Li, B. Construction of Highly Porous and Robust Hydrogen-Bonded Organic Framework for High-Capacity Clean Energy Gas Storage. *Angew. Chem. Int. Ed.* **2024,** *63*, e202411753.
(35) Yang, W.; Greenaway, A.; Lin, X.; Matsuda, R.; Blake, A. J.; Wilson, C.; Lewis, W.; Hubberstey, P.; Kitagawa, S.; Champness, N. R.; Schröder, M. Exceptional Thermal Stability in a Supramolecular Organic Framework: Porosity and Gas Storage. *J. Am. Chem. Soc.* **2010,** *132*, 14457–14469.
(36) Carrera, M.; Such-Basáñez, I.; Marco-Lozar, J. P.; Bueno-López, A.; Vilaplana-Ortego, E.; da Silva, I.; Bautista, D.; Fernández-Alarcón, A.; Calbo, J.; Ortí, E.; Curiel, D. Rational Design of 7-Azaindole-Based Robust Microporous Hydrogen-Bonded Organic Framework for Gas Sorption. *Angew. Chem. Int. Ed.* **2025,** *64*, e202412981.

(37) Lü, J.; Perez-Krap, C.; Suyetin, M.; Alsmail, N. H.; Yan, Y.; Yang, S.; Lewis, W.; Bichoutskaia, E.; Tang, C. C.; Blake, A. J.; Cao, R.; Schröder, M. A Robust Binary Supramolecular Organic Framework (SOF) with High CO_2 Adsorption and Selectivity. *J. Am. Chem. Soc.* **2014,** *136*, 12828–12831.
(38) Furukawa, H.; Yaghi, O. M. Storage of Hydrogen, Methane, and Carbon Dioxide in Highly Porous Covalent Organic Frameworks for Clean Energy Applications. *J. Am. Chem. Soc.* **2009,** *131*, 8875–8883.
(39) Zhang, Y.-B.; Su, J.; Furukawa, H.; Yun, Y.; Gándara, F.; Duong, A.; Zou, X.; Yaghi, O. M. Single-Crystal Structure of a Covalent Organic Framework. *J. Am. Chem. Soc.* **2013,** *135*, 16336–16339.
(40) Yin, Y.; Zhang, Y.; Zhou, X.; Gui, B.; Wang, W.; Jiang, W.; Zhang, Y. B.; Sun, J.; Wang, C. Ultrahigh-Surface Area Covalent Organic Frameworks for Methane Adsorption. *Science* **2024,** *386*, 693–696.
(41) Bracco, S.; Piga, D.; Bassanetti, I.; Perego, J.; Comotti, A.; Sozzani, P. Porous 3D Polymers for High Pressure Methane Storage and Carbon Dioxide Capture. *J. Mater. Chem. A* **2017,** *5*, 10328–10337.
(42) Ben, T.; Pei, C.; Zhang, D.; Xu, J.; Deng, F.; Jing, X.; Qiu, S. Gas Storage in Porous Aromatic Frameworks (PAFs). *Energ. Environ. Sci.* **2011,** *4*, 3991–3999.
(43) Lu, W.; Yuan, D.; Zhao, D.; Schilling, C. I.; Plietzsch, O.; Muller, T.; Bräse, S.; Guenther, J.; Blümel, J.; Krishna, R.; Li, Z.; Zhou, H.-C. Porous Polymer Networks: Synthesis, Porosity, and Applications in Gas Storage/Separation. *Chem. Mater.* **2010,** *22*, 5964–5972.
(44) Yuan, D.; Lu, W.; Zhao, D.; Zhou, H. C. Highly Stable Porous Polymer Networks with Exceptionally High Gas-Uptake Capacities. *Adv. Mater.* **2011,** *23*, 3723–3725.
(45) Lu, W.; Yuan, D.; Zhao, D.; Schilling, C. I.; Plietzsch, O.; Muller, T.; Bräse, S.; Guenther, J.; Blümel, J.; Krishna, R.; Li, Z.; Zhou, H.-C. Porous Polymer Networks: Synthesis, Porosity, and Applications in Gas Storage/Separation. *Chem. Mater.* **2010,** *22*, 5964-5972.
(46) Jia, J.; Chen, Z.; Jiang, H.; Belmabkhout, Y.; Mouchaham, G.; Aggarwal, H.; Adil, K.; Abou-Hamad, E.; Czaban-Jóźwiak, J.; Tchalala, M. R. Extremely Hydrophobic POPs to Access Highly Porous Storage Media and Capturing Agent for Organic Vapors. *Chem* **2019,** *5*, 180–191.
(47) Rozyyev, V.; Thirion, D.; Ullah, R.; Lee, J.; Jung, M.; Oh, H.; Atilhan, M.; Yavuz, C. T. High-Capacity Methane Storage in Flexible Alkane-Linked Porous Aromatic Network

Polymers. *Nat. Energy* **2019,** *4*, 604–611.
(48) Yang, S.; Zhong, Z.; Hu, J.; Wang, X.; Tan, B. Dibromomethane Knitted Highly Porous Hyper-Cross-Linked Polymers for Efficient High-Pressure Methane Storage. *Adv. Mater.* **2024,** *36*, 2307579.
(49) Mason, J. A.; Veenstra, M.; Long, J. R. Evaluating Metal–Organic Frameworks for Natural Gas Storage. *Chem. Sci.* **2014,** *5*, 32–51.
(50) Peng, Y.; Krungleviciute, V.; Eryazici, I.; Hupp, J. T.; Farha, O. K.; Yildirim, T. Methane Storage in Metal-Organic Frameworks: Current Records, Surprise Findings, and Challenges. *J. Am. Chem. Soc.* **2013,** *135*, 11887–94.
(51) Ahmed, A.; Liu, Y.; Purewal, J.; Tran, L. D.; Wong-Foy, A. G.; Veenstra, M.; Matzger, A. J.; Siegel, D. J. Balancing Gravimetric and Volumetric Hydrogen Density in MOFs. *Energ. Environ. Sci.* **2017,** *10*, 2459–2471.
(52) Gómez-Gualdrón, D. A.; Wang, T. C.; García-Holley, P.; Sawelewa, R. M.; Argueta, E.; Snurr, R. Q.; Hupp, J. T.; Yildirim, T.; Farha, O. K. Understanding Volumetric and Gravimetric Hydrogen Adsorption Trade-Off in Metal-Organic Frameworks. *ACS Appl. Mater. Interfaces* **2017,** *9*, 33419–33428.
(53) García-Holley, P.; Schweitzer, B.; Islamoglu, T.; Liu, Y.; Lin, L.; Rodriguez, S.; Weston, M. H.; Hupp, J. T.; Gómez-Gualdrón, D. A.; Yildirim, T.; Farha, O. K. Benchmark Study of Hydrogen Storage in Metal-Organic Frameworks under Temperature and Pressure Swing Conditions. *ACS Energy Lett.* **2018,** *3*, 748–754.
(54) Ahmed, A.; Seth, S.; Purewal, J.; Wong-Foy, A. G.; Veenstra, M.; Matzger, A. J.; Siegel, D. J. Exceptional Hydrogen Storage Achieved by Screening Nearly Half a Million Metal-Organic Frameworks. *Nat. Commun.* **2019,** *10*, 1568–1576.
(55) Chen, Z.; Li, P.; Anderson, R.; Wang, X.; Zhang, X.; Robison, L.; Redfern, L. R.; Moribe, S.; Islamoglu, T.; Gómez-Gualdrón, D. A.; Yildirim, T.; Stoddart, J. F.; Farha, O. K. Balancing Volumetric and Gravimetric Uptake in Highly Porous Materials for Clean Energy. *Science* **2020,** *368*, 297–303.
(56) Wang, J.-X.; Zhang, X.; Jiang, C.; Zhang, T.-F.; Pei, J.; Zhou, W.; Yildirim, T.; Chen, B.; Qian, G.; Li, B. Construction of Highly Porous and Robust Hydrogen-Bonded Organic Framework for High-Capacity Clean Energy Gas Storage. *Angew. Chem. Int. Ed.* **2024,** *63*, e202411753.
